# Hepatitis C Virus Reveals a Novel Early Control in Acute Immune Response

**DOI:** 10.1371/journal.ppat.1002289

**Published:** 2011-10-13

**Authors:** Noëlla Arnaud, Stéphanie Dabo, Daisuke Akazawa, Masayoshi Fukasawa, Fumiko Shinkai-Ouchi, Jacques Hugon, Takaji Wakita, Eliane F. Meurs

**Affiliations:** 1 Institut Pasteur, Hepacivirus and Innate Immunity, Paris, France; 2 National Institute of Infectious Diseases, Department of Virology II, Tokyo, Japan; 3 National Institute of Infectious Diseases, Department of Biochemistry and Cell Biology, Tokyo, Japan; 4 Institut du Fer à Moulin, INSERM UMRS 839, Paris, France; University of California, San Diego, United States of America

## Abstract

Recognition of viral RNA structures by the intracytosolic RNA helicase RIG-I triggers induction of innate immunity. Efficient induction requires RIG-I ubiquitination by the E3 ligase TRIM25, its interaction with the mitochondria-bound MAVS protein, recruitment of TRAF3, IRF3- and NF-κB-kinases and transcription of Interferon (IFN). In addition, IRF3 alone induces some of the Interferon-Stimulated Genes (ISGs), referred to as early ISGs. Infection of hepatocytes with Hepatitis C virus (HCV) results in poor production of IFN despite recognition of the viral RNA by RIG-I but can lead to induction of early ISGs. HCV was shown to inhibit IFN production by cleaving MAVS through its NS3/4A protease and by controlling cellular translation through activation of PKR, an eIF2α-kinase containing dsRNA-binding domains (DRBD). Here, we have identified a third mode of control of IFN induction by HCV. Using HCVcc and the Huh7.25.CD81 cells, we found that HCV controls RIG-I ubiquitination through the di-ubiquitine-like protein ISG15, one of the early ISGs. A transcriptome analysis performed on Huh7.25.CD81 cells silenced or not for PKR and infected with JFH1 revealed that HCV infection leads to induction of 49 PKR-dependent genes, including ISG15 and several early ISGs. Silencing experiments revealed that this novel PKR-dependent pathway involves MAVS, TRAF3 and IRF3 but not RIG-I, and that it does not induce IFN. Use of PKR inhibitors showed that this pathway requires the DRBD but not the kinase activity of PKR. We then demonstrated that PKR interacts with HCV RNA and MAVS prior to RIG-I. In conclusion, HCV recruits PKR early in infection as a sensor to trigger induction of several IRF3-dependent genes. Among those, ISG15 acts to negatively control the RIG-I/MAVS pathway, at the level of RIG-I ubiquitination.These data give novel insights in the machinery involved in the early events of innate immune response.

## Author Summary

Hepatitis C Virus (HCV) is a poor interferon (IFN) inducer, despite recognition of its RNA by the cytosolic RNA helicase RIG-I. This is due in part through cleavage of MAVS, a downstream adapter of RIG-I, by the HCV NS3/4A protease and through activation of the eIF2α-kinase PKR to control IFN translation. Here, we show that HCV also inhibits RIG-I activation through the ubiquitin-like protein ISG15 and that HCV triggers rapid induction of 49 genes, including ISG15, through a novel signaling pathway that precedes RIG-I and involves PKR as an adapter to recruit MAVS. Hence, we propose to divide the acute response to HCV infection into one early (PKR) and one late (RIG-I) phase, with the former controlling the latter. Furthermore, these data emphazise the need to check compounds designed as immune adjuvants for activation of the early acute phase before using them to sustain innate immunity.

## Introduction

IFN induction in response to several RNA viruses involves the intracytosolic pathogen recognition receptor (PRR) CARD-containing DexD/H RNA helicase RIG-I. Following its binding to viral RNA, RIG-I undergoes a change in its conformation through Lys63-type ubiquitination by the E3 ligase TRIM25. This allows its N-terminal CARD domain to interact with the CARD domain of the mitochondria-bound adapter MAVS [1], [2]. MAVS then interacts with TRAF3 to further recruit downstream IRF3 and NF-κB-activating kinases, that stimulate the IFNβ promoter in a cooperative manner. In addition, IRF3 stimulates directly the promoters of some interferon-induced genes (early ISGs) while NF-κB stimulates that of inflammatory cytokines [3].

The RNA of Hepatitis C virus (HCV) has an intrinsic ability to trigger IFNβ induction through RIG-I [4], [5], [6]. Yet HCV is a poor IFN inducer. One reason for this comes from the ability of its NS3 protease to cleave MAVS [7]. Another relates to the ability of HCV to trigger activation of the dsRNA-dependent eIF2α kinase PKR [8], [9] which leads to inhibition of IFN expression through general control of translation while the viral genome can be translated from its eIF2α-insensitive IRES structure [8].

HCV infection can trigger important intrahepatic synthesis of several IFN-induced genes (ISGs) in patients [10], [11] and in animal models of infection in chimpanzees [12]. Expression of ISGs can be explained at least in part by the ability of HCV to activate the IFN-producing pDCs in the liver through cell-to-cell contact with HCV-infected cells [13]. Intriguingly, despite the recognized antiviral activity of a number of these ISGs, their high expression paradoxically represents a negative predictive marker for the response of these patients to standard combination IFN/ribavirin therapy [14], [15], [16]. The ubiquitine-like protein ISG15 is among the ISGs which are the most highly induced by HCV [16] and was recently shown to act as a pro-HCV agent [17]. Interestingly, ISG15 was also shown to control RIG-I activity through ISGylation [18].

Here, we show that HCV controls IFN induction at the level of RIG-I ubiquitination through the ubiquitine-like protein ISG15, one of the early ISGs. Use of small interfering RNA (siRNA) targeting to compare the effect of ISG15 to that of PKR on IFN induction and HCV replication led to the unexpected finding that HCV infection triggers induction of ISG15 and other ISGs by using PKR as an adapter through its N terminal dsRNA binding domain. This recruits a signaling pathway which involves MAVS,TRAF3 and IRF3 but not RIG-I. Altogether, our results present a novel mechanism by which HCV uses PKR and ISG15 to attenuate the innate immune response.

## Results

### HCV infection negatively controls RIG-I ubiquitination

**Figure 1:**
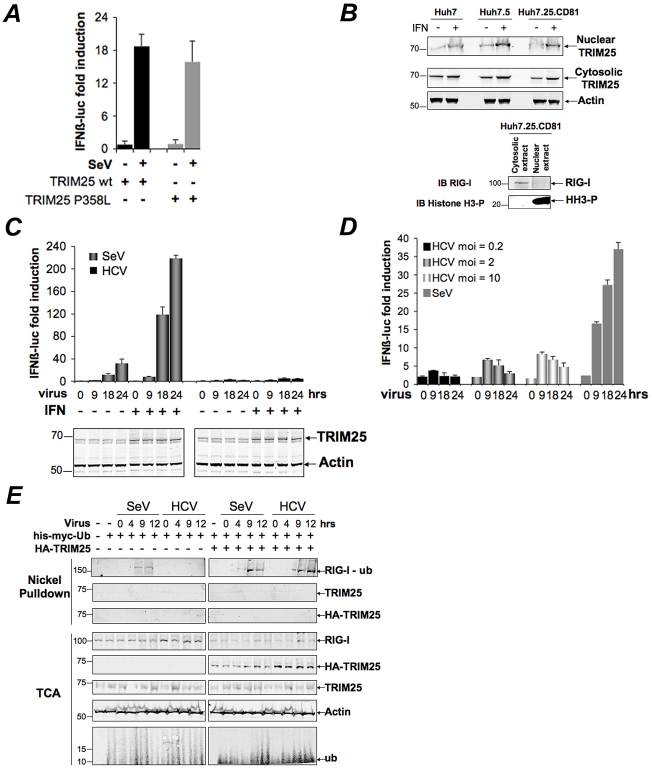
HCV infection negatively controls RIG-I ubiquitination. (**A**) Huh7.25.CD81 cells were transfected for 24 hrs with 150 ng of the pGL2-IFNβ-FLUC; 40 ng of the pRL-TK-RLUC reporter plasmids alone or in presence of 150 ng of a plasmid expressing HA-TRIM25, either as such (TRIM25wt) or containing the P_358_L substitution (a SNP rs75467764 with no reported pathology). Cells were infected or not with SeV (40 HAU/ml) for 24 hrs. IFN expression was expressed as fold induction of luciferase activity. Error bars represent the mean ± S.D. for triplicates. (**B**) Huh7, Huh7.5 and Huh7.25.CD81 cells were either untreated or treated with 500 U/ml IFNα for 24 hrs. TRIM25 was detected by immunoblot after preparation of nuclear and cytosolic fractions from 25 µg of cell extracts. Detection of Actin, RIG-I and phosphorylated Histone 3 (HH3-P) served as controls. (**C–D**) Huh7.25.CD81 cells were transfected with the reporter plasmids as in A, a few hours before being treated with 500 U/ml IFNα for 24 hrs (**C**) or left untreated (**C or D**). They were then infected with Sendai virus (40 HAU/ml) or with JFH1 at an m.o.i of 0.2 (**C**) or increasing from 0.2 to 10 (**D**). At the times indicated, IFN expression was expressed as fold induction of luciferase activity. Error bars represent the mean ± S.D. for triplicates. Induction of TRIM25 after IFN treatment was shown by immunoblot (**C**). (**E**) The Huh7.25.CD81 cells were transfected for 48 hrs with 5 µg of His-Myc-Ubiquitin expression plasmid in absence or presence of a plasmid expressing HA-TRIM25 and infected with SeV (40 HAU/ml) or HCV (m.o.i = 6). At the times indicated, 10% of the lysate was precipitated with TCA and the remaining lysate subjected to nickel pulldown under denaturing conditions. Total and ubiquitin (Ub)-modified proteins were separated by SDS-PAGE and revealed by immunoblot.

We recently reported that the HCV permissive Huh7.25.CD81 cells [19] that we used to identify the pro-HCV action of PKR, did not induce IFN in response to HCV infection, unless after ectopic expression of TRIM25 [8]. We started this study by investigating at which level this defect could occur. A P_358_L substitution in the endogenous TRIM25 of these cells, revealed by sequence analysis, proved to have no incidence of the ability of TRIM25 to participate in the IFN induction process. Indeed, ectopic expression of a TRIM25 P_358_L construct was as efficient as a TRIM25wt construct to increase IFN induction in the Huh7.25.CD81 cells, after infection with Sendai virus (SeV) (**Figure 1A**). Like some other members of the TRIM family, TRIM25 is localized in both the cytosol and nucleus and is induced upon IFN treatment [20]. No specific difference between the cellular localization of TRIM25 was observed in the Huh7.25.CD81 cells when compared to Huh7 cells or Huh7.5 cells, which rules out a role for a cellular mislocalization in its inability to participate in IFN induction (**Figure 1B**). TRIM25 was also efficiently induced by IFN (**Figure 1B** **and Figure S1**). We assayed whether increasing TRIM25 upon IFN treatment could mimic the effect of its ectopic expression and restore IFN induction in response to HCV infection. However, this resulted only in a poor stimulation of an IFNβ promoter (3 to 5-fold), in contrast to its effect upon SeV infection (230-fold) (**Figure 1C**). Similarly, HCV infection at higher m.o.i, as an attempt to favour recognition of RIG-I by the viral RNA, only modestly increased IFN induction (**Figure 1D**). TRIM25 plays an essential role in IFN induction through RIG-I ubiquitination [1]. We then analysed whether this step was affected by HCV infection in the Huh7.25.CD81 cells. The results showed that, in contrast to SeV infection used as control, HCV infection could not trigger RIG-I ubiquitination, unless the cells are supplied with ectopic TRIM25 (**Figure 1E**). Thus, HCV infection appears to mediate a control on IFN induction through regulation of RIG-I ubiquitination.

### HCV controls RIG-I ubiquitination through ISG15

**Figure 2:**
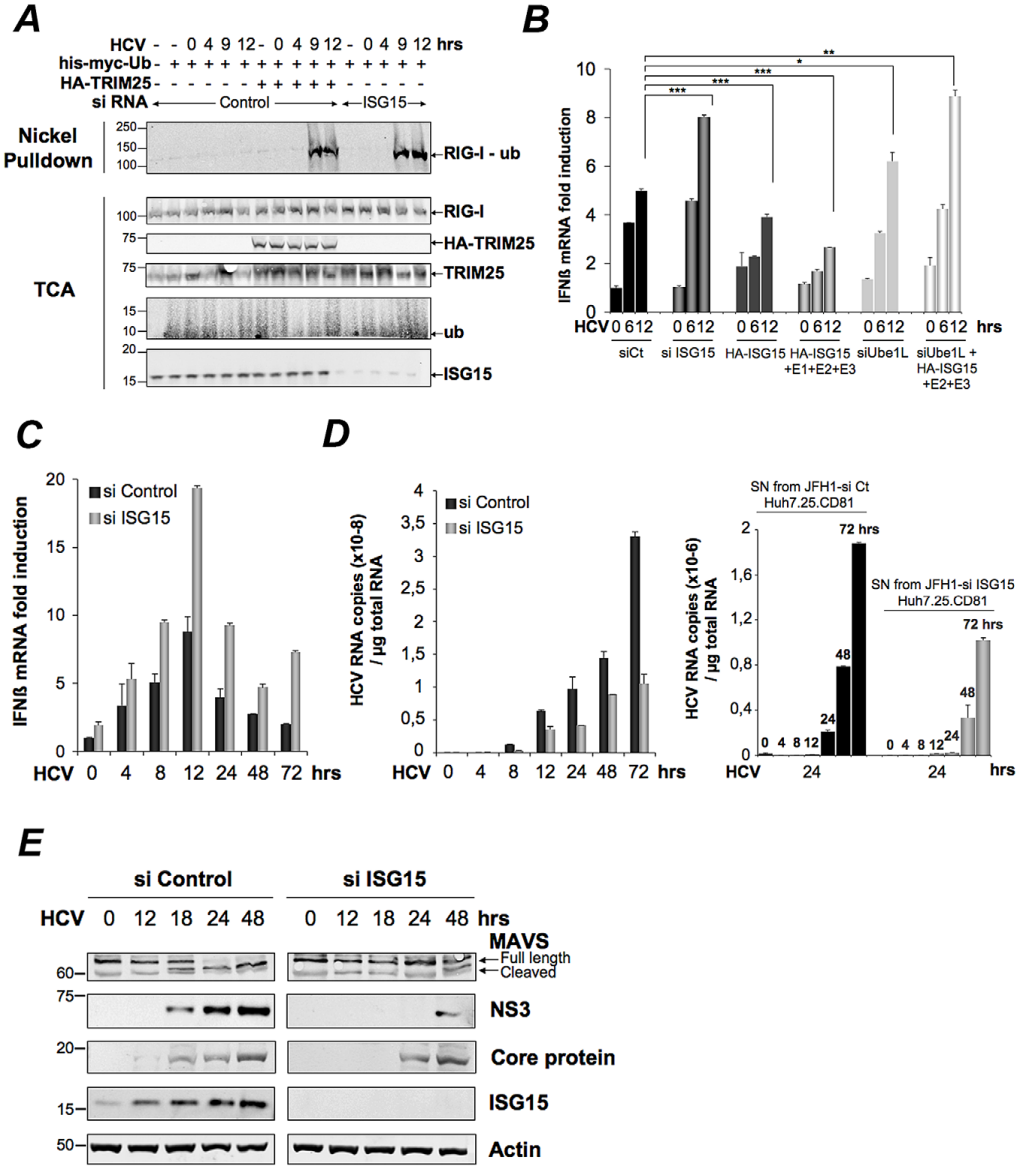
HCV controls RIG-I ubiquitination through ISG15. (**A**) Huh7.25.CD81 cells were transfected for 24 hrs with 25 nM of siRNA (Control or ISG15) and for another 24 hr with 5 µg of a His-Myc-Ubiquitin plasmid in absence or presence of 5 µg of a plasmid expressing HA-TRIM25. The cells were infected with JFH1 (m.o.i = 0.2). At the times indicated, cell extracts were processed for analysis of RIG-I ubiquitination and the expression of the different proteins in the total cell extracts. (**B**) Huh7.25.CD81 cells were first transfected with siRNA Control (25 nM), si RNA ISG15 (25 nM), siRNA Ube1L (50 nM) or left untreated. After 24 hrs, the untreated cells were transfected with a plasmid expressing HA-ISG15 (500 ng) alone or in presence of plasmids expressing E1, E2 and E3 (1 µg each) while a set of cells transfected with siRNA Ube1L received plasmids expressing HA-ISG15, E2 and E3. After 24 hrs, the cells were infected with JFH1 (m.o.i = 6) for the times indicated. Stimulation of endogenous IFNβ RNA expression was determined by RTqPCR and expressed as fold induction. The degree of statistical significance is indicated by stars after calculation of the p-values (from left to right: 0.0005, 0.0076, 0.0003, 0.047 and 0.0023). (**C–D**) Huh7.25.CD81 cells, transfected with 25 nM of siRNA (Control or ISG15) for 48 hrs, were infected with JFH1 (m.o.i = 6) for the times indicated. Expression of IFNβ or HCV RNA, determined by RTqPCR, was expressed as fold induction (**C**; IFNβ) or as copies (**D**; HCV). Error bars represent the mean ±S.D for triplicates. Expression levels of IFNβ RNA at the start of infection were 2.1×10^4^ (siControl) and 4×10^4^ copies (siISG15). Supernatants collected at different times post-infection were used to infect fresh cells. After 24 hours, the RNAs were extracted from the cells and expression of HCV RNA was determined by RTqPCR. (**E**) Huh7.25.CD81 cells, transfected with 25 nM of siRNA (Control or ISG15) for 48 hrs, were infected with JFH1 for the times indicated. Cell extracts were analysed by immunoblot with Abs directed against ISG15, MAVS, the HCV NS3 and core proteins and Actin as loading control.

Inhibition of the function of TRIM25 or RIG-I ubiquitination has been suggested to occur via the small ubiquitin-like protein ISG15 and the process of ISGylation [18], [21]. We then analysed whether ISG15 was involved in the control of RIG-I ubiquitination upon HCV infection. For this, we chose a transient transfection approach using siRNAs targeting ISG15 in the Huh7.25.CD81 cells. Indeed, this resulted in a strong ubiquitination of RIG-I at 9 hrs and 12 hrs post-HCV infection, which was equivalent to that observed in cells supplied with ectopic TRIM25 (**Figure 2A**). A similar result was obtained after JFH1 infection in the Huh7 cells, used as another HCV-permissive cell line (**Figure S2**). Thus, ISG15 can control RIG-I ubiquitination in different cells infected by HCV. We next investigated whether ISGylation was involved in this process. Absence of detection of RIG-I ubiquitination after HCV infection of the Huh7.25.CD81 cells precludes direct analysis of the effect of ISG15 on RIG-I. We used an IFNβ-luc reporter assay instead, as it proved to be sensitive enough to detect some IFN induction in response to JFH1 infection in those cells (see Figure 1D). We found that IFN induction increased when cells were transfected with siRNAs targeting ISG15 while it decreased in cells overexpressing IGS15 (**Figure 2B**). Expression of ISG15 in the presence of the E1, E2 and E3 ligases involved in ISGylation (respectively Ube1L, UbcH8 and HERC5) [22] further inhibits IFNβ induction (**Figure 2B**). Similar results were observed upon infection with Sendai virus (**Figure S3**). The ISGylation process is strictly dependent on the presence of the E1 ligase Ube1L [23]. Indeed, enhanced IFN promoter activity has been observed in Ube1L−/− cells in response to NDV [18]. In accord with this, depletion of endogenous Ube1L from the Huh7.25.CD81 cells (**Figure S4**), as such or after ectopic expression of ISG15, UbcH8 and HERC5, resulted in an increase in IFNβ induction after infection with HCV (**Figure 2B**). We then analysed the effect of siISG15 on IFNβ induction after infecting the cells with HCV up to 72 hours, in order to pass through the 24 hr time-point where the signaling pathway leading to the transcription of this gene is expected to stop because of the NS3/4A-mediated cleavage of MAVS [8]. The results show that, whereas IFNβ transcription was indeed strongly inhibited after 24 hr in the control cells, it still occurred significantly in the cells expressing siRNA ISG15 (**Figure 2C**). Previous data have shown a positive role for ISG15 on HCV production [24], [25]. In accord with this, silencing of ISG15 resulted in clear inhibition of HCV RNA expression with however no significant consequence on the ability of the virions produced to re-infect fresh cells (**Figure 2D**). Analysis of expression of MAVS and NS3, as well as the expression of the core protein as another example of viral protein, then showed that the depletion of ISG15 both decreased and delayed the expression of the viral proteins as compared to the siRNA control cells and that this was correlated by a delay in the NS3/4A-mediated cleavage of MAVS (**Figure 2E**). These results show that ISG15 controls the process of IFN induction during HCV infection by interfering with RIG-I ubiquitination through an ISGylation process and by boosting efficient accumulation of NS3, among other viral proteins, thus favouring its negative control on IFN induction by cleavage of MAVS.

### ISG15 strengthens the pro-HCV activity of PKR

**Figure 3:**
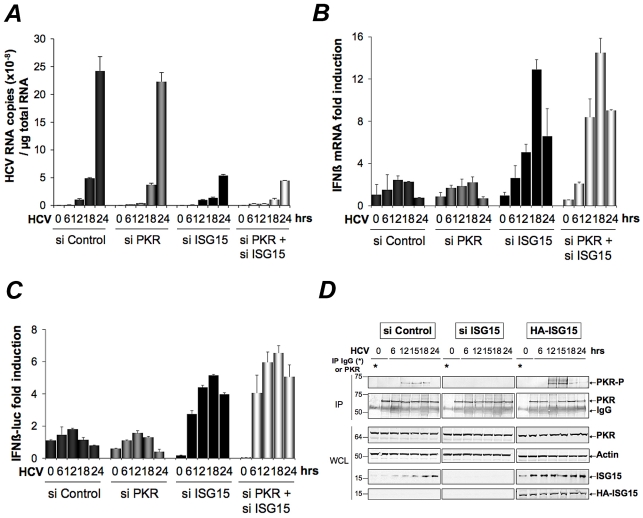
ISG15 strengthens the pro-HCV activity of PKR. (**A–B**). The Huh7.25.CD81 cells were transfected with 25 nM of the different siRNA (Control, ISG15, PKR), separately or together. After 48 hrs, cells were infected with JFH1 (m.o.i = 0.2). At the times indicated, expression of HCV or IFNβ RNA was determined by RTqPCR and expressed as copies of JFH1 RNA (**A**) or as fold induction (IFNβ; **B**). The expression levels of IFNβ RNA at the start of infection was 6.96×10^5^ copies. (**C**) Two sets of Huh7.25.CD81 cells were first transfected with siRNA ISG15, siRNA PKR separately and together for 24 hrs, then transfected with the reporter plasmids IFNβ-firefly luciferase (pGL2-IFNβ), pRL-TK Renilla-luciferase for another 24 hrs and infected with JFH1 (m.o.i = 0.2) for the times indicated. In each case, IFN expression was expressed as fold-induction over control cells that were simply transfected with pGL2-IFNβ-FLUC/pRL-TK-RLUC. The graph represents the level of firefly luciferase activity normalized to the ratio R-luc RNA/GAPDH RNA. Such normalization is required because of the negative control of general translation through PKR after 12 hrs post-infection [8]. Error bars represent the mean ±S.D for triplicates. (**D**) Huh7.25.CD81 cells, in 100 cm^2^ plates, were transfected with siRNA Control or siRNA ISG15 or transfected with a plasmid expressing HA-ISG15 for 48 hrs and infected with JFH1 (m.o.i = 6). At the indicated times post-infection, cell extracts (2.2 mg) were processed for immunoprecipitation of PKR or for incubation with mouse IgG as a control of specificity (asterisk). The immunoprecipitated complexes were run on two different NuPAGE gels and blotted using Mab 71/10 or anti-phosphorylated PKR antibodies (PKR-P). The presence of PKR and PKR-P was revealed using the Odyssey procedure. The ratio PKR-P/PKR in the absence or in the presence of ISG15, either endogenous or endogenous and ectopic, is shown in Figure S5.

ISG15 ([24], [25] and this study) and PKR [8], [9] emerge as two ISGs with pro-HCV activities, instead of playing an antiviral role. We then assayed the effect of a combined depletion of PKR and ISG15 on HCV replication and IFN expression in the Huh7.25.CD81 cells. As shown in Figure 2 D and B, siRNAs targeting ISG15 were sufficient both to inhibit HCV replication (**Figure 3A**) and to increase IFNβ expression, either measured by RTqPCR (**Figure 3B**) or by using an IFNβ-luciferase reporter assay (**Figure 3C**). Very limited additional effect was observed in the concomitant presence of siRNAs targeting PKR. (**Figure 3B**). Interestingly, we noticed that expression of luciferase from the IFNβ promoter increased throughout the first 18 hours of HCV infection in the siISG15 cells (**Figure 3C**). This was intriguing as it should have been inhibited after 12 hours of HCV infection through the eIF2α kinase activity of PKR and its control on translation [8]. We therefore analysed whether the state of PKR activation (phosphorylation) was dependent on the expression of ISG15. For this, the Huh7.25.CD81 cells were transfected either with siRNAs targeting ISG15 or with a plasmid expressing an HA-ISG15 construct and PKR phosphorylation was analysed as described previously [8]. The results showed that depletion of ISG15 inhibits PKR activation in the HCV-infected cells, while its overexpression stimulates it (**Figure 3D** and **Figure S5**). Therefore these data reveal that, in addition to negatively controlling RIG-I ubiquitination, ISG15 can also positively control PKR activity. The conjugation of both effects results in an efficient control of IFN induction during HCV infection.

### HCV triggers a PKR-dependent pathway early in infection to induce ISG15 and other genes

**Figure 4:**
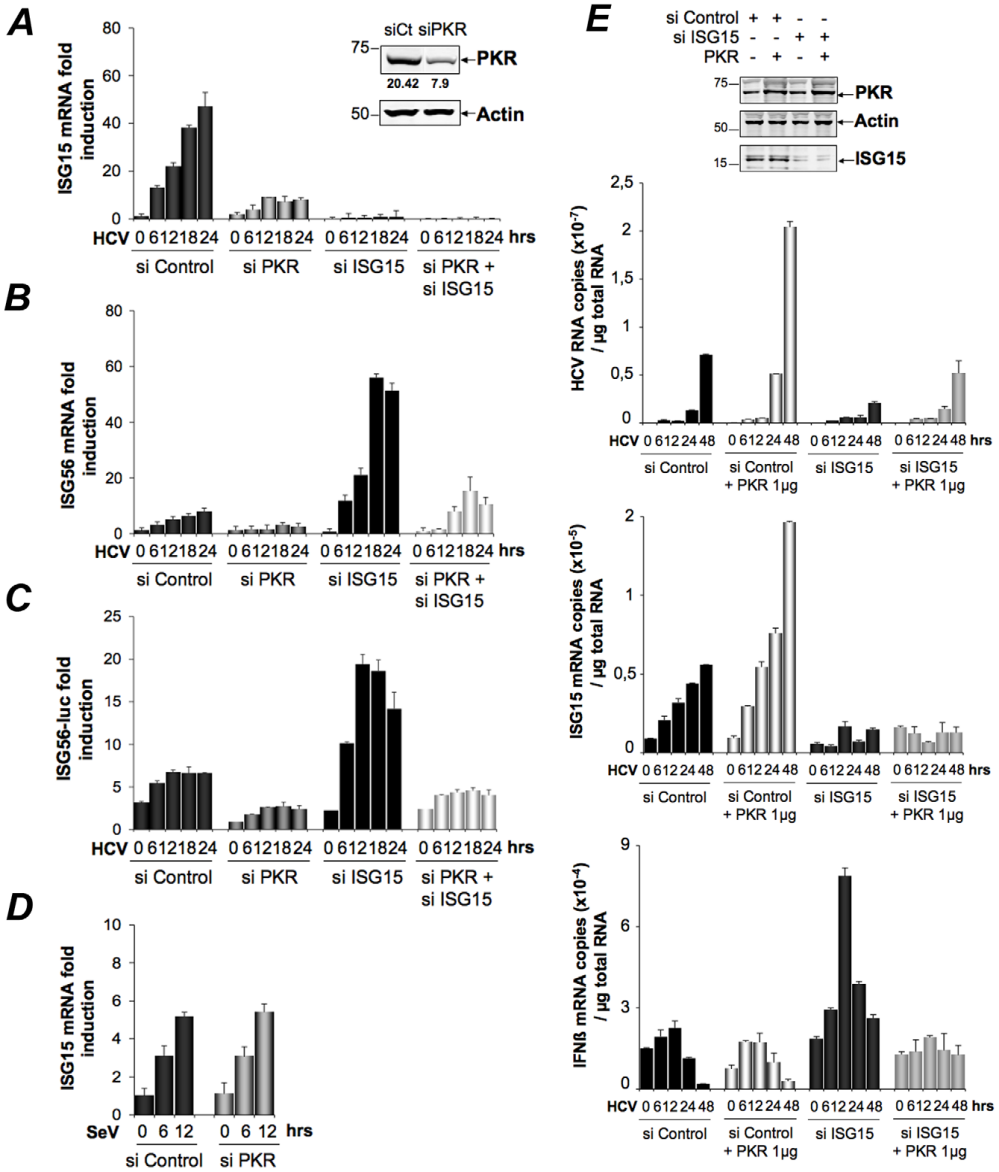
HCV triggers a PKR-dependent pathway early in infection to induce ISG15 and other genes. (**A–C**) The cDNAs reversed transcribed from the RNAs extracted from the Huh7.25.CD81 cells for the experiment described under Figure 3A were analysed by qPCR for the expression of ISG15 (**A**) and ISG56 (**B**). Expression levels of ISG15 and ISG56 RNA at the start of infection were respectively 1×10^5^ and 1.18×10^5^ copies. A novel set of Huh7.25.CD81 cells were transfected with siRNA Control, siRNA ISG15, siRNA PKR separately and together for 48 hrs. They were then transfected with the reporter plasmids ISG56-FLUC and pRL-TK-RLUC and infected with JFH1 (m.o.i = 0.2). At the times indicated, the effect of the different conditions of silencing on the reporter expression was analyzed after normalization performed as described under Figure 3C (**C**). Results are expressed as fold induction. Error bars represent the mean ±S.D for triplicates. (**D**) Huh7.25.CD81 cells were either transfected with 25 nM of siRNA Control or siPKR for 24 hrs and infected with SeV for the times indicated. Expression of endogenous ISG15 was determined by RTqPCR and expressed as fold induction. Error bars represent the mean ±S.D for triplicates. The expression levels of ISG15 RNA at the start of infection were respectively 4.91×10^4^ copies (siControl) and 5.44×10^4^ copies (siPKR). (**E**) Huh7.25.CD81 cells were either transfected with 25 nM of siRNA Control or siISG15 and with 1 µg of a plasmid expressing PKR where indicated. After 48 hrs, the cells were infected with HCV (m.o.i = 6) for the times indicated. Expression of HCV, ISG15 and IFNβ RNA was determined by RTqPCR. Cell lysates prepared from cells treated in the same conditions but not infected were used to control expression of PKR and ISG15 by immunoblot.

The Huh7.25.CD81 cells express ISG15 at significant basal levels. This situation was not surprising as various cellular systems can also express some of the ISGs at basal level. Expression of ISG15 was approximately 2- and 5-fold higher in the Huh7.25.CD81 cells than in the Huh7.5 or Huh7 cells (data not shown). Intriguingly however, we noticed that ISG15 expression was increased in response to HCV infection (see **Figure 2E**). To investigate this further, we simply re-used the RNAs prepared for the experiment shown in Figure 3B and performed a quantitative kinetics analysis. The results confirmed that HCV can trigger induction of ISG15 (**Figure 4A**). Unexpectedly, analysis of the RNA extracted from the cells treated with siRNAs targeting PKR, revealed that ISG15 RNA expression was strongly repressed when PKR was silenced (**Figure 4A**). This surprising result was confirmed by analysing induction of ISG56, another early ISG [26], both at the level of its endogenous RNA (**Figure 4B**) or by using an ISG56-luciferase vector (**Figure 4C**). In the latter case, a strong increase of the reporter expression in the cells treated with siRNAs targeting ISG15, was similar to the situation observed for IFNβ RNA (**Figure 2B and 2C**). This can be related to activation of the RIG-I pathway, which can function when ISG15 is absent. These data suggest that HCV may use PKR to activate gene transcription. Importantly, this phenomenon was specific to HCV as infection with Sendai virus resulted in a similar induction of ISG15 and ISG56, regardless of PKR (**Figure 4D** **and Figure S6**). We then examined whether overexpression of PKR could boost induction of ISG15 during HCV infection and how this would affect HCV replication and IFN induction, in relation to the pro-HCV action of ISG15. Huh7.25.CD81 cells were transfected with a plasmid expressing PKR alone or in presence of siRNAs targeting ISG15, before being infected with HCV over 48 hours. Overexpression of PKR increased the ability of HCV to induce ISG15 and concomitantly, led to an increase in HCV RNA expression. The latter increase was abolished when ISG15 was silenced, thus showing that the PKR-dependent increase in HCV expression is mediated by ISG15 (Figure **4E**). However, while the cells silenced for ISG15 are able to induce IFN in response to HCV infection, as shown in Figure 3B, they are unable to do so when PKR is overexpressed. This suggests that PKR may also interfere with the process of IFN induction, independently of ISG15, a possibility that remains to be explored.

A role for PKR in gene induction in response to HCV infection has not been described before. Additional information was therefore obtained through a transcriptome analysis of 2165 genes in the Huh7.25.CD81 cells treated with control siRNAs or siRNAs targeting PKR and infected with HCV for 12 hrs. Out of the most significant 422 genes that were identified, 99 were unmodified or barely modified and 33 were down-regulated, while 290 genes were found to be up-regulated by HCV infection (data not shown). Among those, HCV infection triggered up-regulation of 49 genes which are directly dependent on PKR expression (**Table 1**). Forty percent of these genes (20) belong to the family of the ISGs, with ISG15 among the most induced genes (**Table 1**). In the reciprocal situation, only 17 genes depended on PKR for their down-regulation by HCV infection, with no link to a particular family of genes and limited variation both in number and intensity (**Table S1**). Thus, induction of ISGs upon HCV infection may occur through a novel signaling pathway that involves PKR.

**Table 1:** PKR-dependent up-regulated genes upon HCV infection.

SiPKR mock/siCt	Name	Access. N.	siCtMock	siCt HCV	siCtMock'	siPKRHCV	LOG2*
0,6	**ISG56**	NM_001548	15,0	885,8	10,2	7,6	-6,3
0,7	**ISG15**	NM_005101	593,6	26061,9	410,6	283,5	-6,0
0,7	**IFI 9-27/IFITM1**	NM_003641	27,8	817,7	15,1	10,1	-5,5
1,2	**IFI1-8U**	NM_006435	24,0	597,7	10,9	7,2	-5,3
1,1	Olfactory Receptor 9l1	NM_001005211	26,6	473,1	10,1	4,8	-5,2
1,6	IFI1-8U	XM_084845	17,7	365,4	9,3	6,5	-4,9
0,8	**OASp100**	NM_006187	46,4	909,9	40,0	33,5	-4,5
0,8	**IFI6-16**	NM_002038	834,5	10040,6	45,9	24,1	-4,5
0,6	**Ub2L6**	NM_004223	392,7	4078,9	281,2	128,7	-4,5
0,9	**OAS 1**	NM_016816	49,9	704,03	31,8	21,6	-4,4
0,9	**ISG12**	NM_005532	46,3	592,54	38,3	29,2	-4,1
0,8	**IFP 35**	NM_005533	36,3	369,7	26,6	16,9	-4,0
0,6	PARP-9	NM_031458	29,5	318,5	37,8	25,6	-4,0
0,5	GABA-B receptor 1	NM_006398	29,5	500,8	26,0	28,5	-4,0
0,7	**Lysp100B**	NM_003113	8,7	93	8,5	6,1	-3,9
0,8	PDIP1	NM_033405	27,4	146,6	28,0	12,8	-3,6
0,8	**PKR**	NM_002759	48,2	306,8	47,0	26,0	-3,5
1,6	**MT-IM**	NM_176870	49,0	1371,8	6,9	19,6	-3,3
0,7	**Phospholipid scramblase**	NM_021105	170	1137,2	189,9	153	-3,1
1,1	**RIG-I**	NM_014314	23,5	223,2	18,9	21,9	-3,0
0,6	**IFIT-5**	NM_012420	24,9	95,3	35,0	21,3	-2,7
1,3	RIG-I	NM_004585	7,4	42,5	6,3	6,0	-2,6
0,7	**STAT1 beta**	NM_139266	336,9	1401,5	300,3	210,3	-2,6
0,8	BRCA1 C-ter assoc. Prot	NM_001040444	12,3	45,1	8,1	5,1	-2,6
0,9	Cohesin Rec8 homolog	NM_005132	18,0	103	16,7	16,9	-2,5
0,5	C/EBPdelta	NM_005195	324,9	901,8	278,9	161,27	-2,3
0,7	ZNF532	NM_018181	32,8	146,5	25,5	23,8	-2,3
0,6	NNMT	NM_006169	52,8	143,8	50,26	28,9	-2,2
1	ISG1-8U	XM_084845	32,0	146,8	23,1	22,7	-2,2
1,1	HIF00	NM_153833	45,3	199,9	34,7	32,9	-2,2
0,8	**ISG20**	NM_002201	129,3	338,3	107,5	61,4	-2,2
1,1	PSMB10	NM_002801	16,2	75,1	14,5	15,0	-2,2
1,3	ZC3HAV1	NM_024625	8,3	26,0	6,9	4,9	-2,1
0,9	SOD2	NM_000636	348,5	1612,2	311	334,3	-2,1
0,7	PARP12	NM_022750	269,9	875,2	296,9	224,2	-2,1
0,7	NMI	NM_004688	32,1	136	37,4	37,0	-2,1
0,8	NEDD9	NM_006403	5,7	19,0	5,7	4,7	-2,0
1,1	**SAMHD1**	NM_015474	17,1	49,5	13,6	9,7	-2,0
0,7	AKT2	NM_001626	13,4	20,0	14,2	5,4	-2,0
0,5	ARG1	NM_000045	245,9	282,6	231,4	67,0	-2,0
0,8	BHLHB2	NM_003670	76,4	128,0	71,5	30,5	-2,0
0,8	LGALS3BP	NM_005567	22,0	72,0	16,1	13,6	-2,0
1,3	ZNF292	XM_048070	22,3	31,4	20,2	7,3	-2,0
1,1	STAT1	NM_007315	53,3	275,3	48,2	64,9	-1,9
0,7	TBA3_HUMAN	NM_006009	28,2	43,6	33,4	13,7	-1,9
0,5	TM4SF20	NM_024795	45,2	51,0	36,9	11,1	-1,9
1,4	ERAP2	NM_022350	9,8	19,2	8,8	4,6	-1,9
0,8	**USP18**	XM_001126794.1	215,1	979,2	201,8	245,9	-1,9
1	USP18	XM_001126794.1	129,6	617,0	127,3	165,5	-1,9

The Huh7.25.CD81 cells, seeded at 3.10^6^ cells in 10-cm plates, were transfected after 24 hrs with 25 nM of siRNA Control or siRNA PKR using Fugene HD. 24 hrs post transfection, they were either mock-infected or infected for 2 hrs at 37°C with JFH1 (moi = 0.2) (three independent plates/sample). The medium was then removed and cells were incubated with complete DMEM for 12 hrs at 37°C. The cells were washed twice with TBS containing phosphatase and protease inhibitors, harvested by scraping, the cell pellets were centrifuged, the supernatants were removed and the pellets were frozen and stored at −80°C before being processed for micro-array.The list shows genes that were affected no more than twice by the depletion of PKR in the control cells (0.5< siPKR mock/siCt <1.6). The dependence of each of these genes in regards with PKR for their induction by HCV is expressed as log2 (ratio (siPKR HCV/siCt Mock)−(siCt HCV/siCt Mock) (indicated by log2*) with a cut-off of ≈2.0 fold.

### Induction of ISG15 by HCV is independent of RIG-I, involves MAVS/TRAF3 association with PKR and involves the DRBD region but not the catalytic activity of PKR

**Figure 5:**
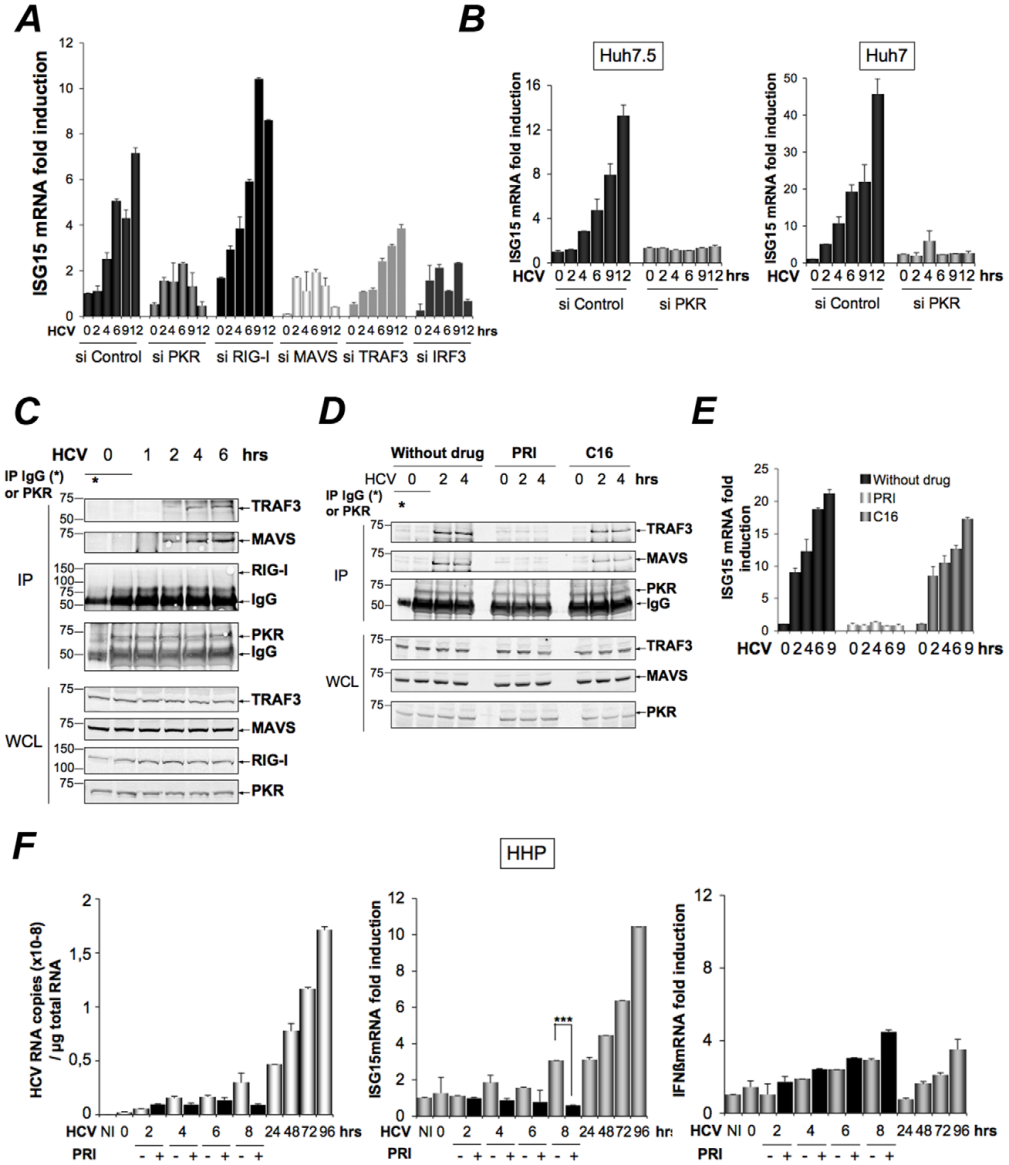
HCV-dependent induction of ISG15 involves PKR, MAVS and TRAF3 and not RIG-I. (**A–B**). **A**-The Huh7.25.CD81 cells were transfected with 50 nM Control siRNA and the different Smartpool siRNAs (50 nM siPKR; 10 nM siRIG-I; 5 nM siMAVS; 50 nM siTRAF3; 50 nM siIRF3) for 48 hrs and infected with JFH1 (m.o.i = 6). (**B**) Huh7.5 or Huh7 cells were transfected with siRNA Control or siPKR (50 nM) for 48 hrs and infected with JFH1 (m.o.i = 0.2 for Huh7.5 or 10 for Huh7). At the times indicated, expression of endogenous ISG15 was determined by RTqPCR and expressed as fold induction. Error bars represent the mean ±S.D for triplicates. The expression level of ISG15 RNA at the start of infection in the siControl cells was 9.97×10^4^ copies (Huh7.25.CD81), 1.31×10^4^ copies (Huh7.5) and 1.28×10^4^ (Huh7). (**C–D**) Huh7.25.CD81 cells, in 100 cm^2^ plates, were infected with JFH1 (m.o.i = 0.2) alone (**C**) or in presence of PRI or C16 (**D**). At the times indicated, cell extracts (3.5 mg) were processed for immunoprecipitation of PKR or for incubation with mouse IgG as a control of specificity (asterisk). The detection of the proteins in the complexes and in the whole cell extracts (WCE) was revealed by immunoblot using the Odyssey procedure. (**E**) The Huh7.25.CD81 cells were incubated with PRI or C16 and infected with JFH1 (m.o.i = 0.2) for the times indicated. Expression of endogenous ISG15 was determined as in A–B. The ISG15 RNA levels were 3.81×10^4^ copies in the siControl cells. (**F**) Human primary hepatocytes (HHP) were infected with JFH1 (m.o.i = 6). One set of cells was incubated with 30 mM of the PRI inhibitor during 8 hours. At the times indicated, expression of HCV RNA, ISG15 and IFNβ was determined by RTqPCR. The expression levels of ISG15 and IFNβ RNA at the start of infection was 1.05×10^5^ copies and 1,11×10^4^ copies, respectively. Inhibition of induction of ISG15 by PRI at 8 hr post-infection was statistically significant (***; p = 0.0001).

Infection with RNA viruses or transient transfection with dsRNA can directly and rapidly induce early ISGs, such as ISG15, through IRF3, after activation of the RIG-I/MAVS pathway and recruitment of TRAF3, an essential adapter which recruits the downstream IRF3 kinases TBK1/IKKε. We have shown that the RIG-I pathway was not operative during HCV infection in the Huh7.25.CD81 cells, precisely due to the presence of ISG15. To determine how ISG15 induction through PKR relates to or differs from the RIG-I/MAVS pathway, the Huh7.25.CD81 cells were treated with siRNAs aimed at targeting separately PKR, RIG-I, MAVS, TRAF3 and IRF3 (**Figure S7**) and infected with HCV. The results clearly showed that induction of ISG15 in response to HCV infection depends on PKR, MAVS, TRAF3 and IRF3 but not on RIG-I (**Figure 5A**). The participation of IRF3 was further confirmed by immunofluorescence studies which showed its nuclear translocation at 6 hours post-infection (**Figure S8**). ISG15, as well as ISG56, was also clearly induced in response to HCV infection in two other HCV permissive cell lines, such as Huh7 and Huh7.5 cells, and this induction was abrogated in presence of siRNAs targeting PKR (**Figure 5B** **and Figure S9**). Importantly, since Huh7.5 cells express a non-functional RIG-I/MAVS pathway due to a mutation in RIG-I, result with these cells supports the notion that the ability of HCV to trigger induction of ISGs through PKR is independent of RIG-I. To have more insights on this novel PKR signaling pathway, PKR was immunoprecipitated at early times points following infection of Huh7.25.CD81 cells with HCV and the immunocomplexes were analysed for the presence of MAVS, TRAF3 and RIG-I. Both MAVS and TRAF3, but not RIG-I, associate with PKR in a time dependent manner, beginning at 2 hrs post-infection (**Figure 5C**). Strikingly, these associations were abrogated by the cell-permeable peptide PRI which is analogous to the first dsRNA binding domain (DRBD) of PKR [8], while unaffected by C16, a chemical compound which inhibits the catalytic activity of PKR (**Figure 5D**). In line with this, PRI but not C16, abrogated the ability of HCV to induce ISG15 (**Figure 5E**). The same result was obtained for induction of ISG56 (**Figure S10**). We then used human primary hepatocytes (HHP) to determine whether HCV was also able to induce ISGs through PKR in a more physiological cellular model. A follow-up of the infection over a period of 96 hours showed that JFH1 was replicating correctly in those cells as well as leading to induction of ISG15 (10-fold) and to some induction of IFNβ (2.5-fold). These cells were infected with JFH1 for 8 hours in the absence or presence of PRI, making convenient use of the cell-penetrating ability of this peptide. Longer period of treatment with PRI were not investigated for practical reasons (see Materials and Methods). The results showed that PRI was significantly inhibiting the induction of ISG15 while it had no effect on that of IFNβ (**Figure 5F**). Altogether, these data demonstrate that HCV triggers induction of early ISGs through MAVS and TRAF3 by using PKR as an adapter protein.

### PKR interacts both with MAVS and TRAF3 and binds HCV RNA ahead of RIG-I

**Figure 6:**
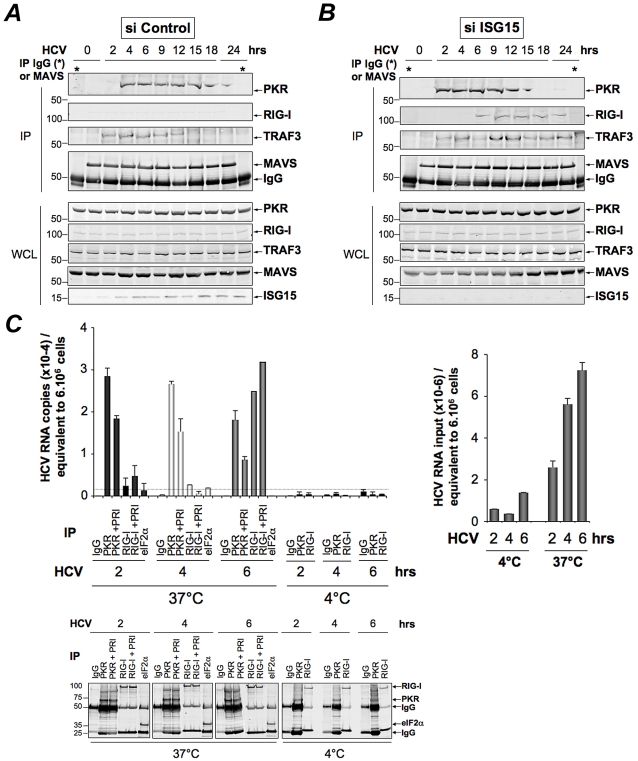
PKR both interacts with MAVS and TRAF3 and binds HCV RNA ahead of RIG-I. (**A–B**)- Huh7.25.CD81 cells were transfected with 25 nM of siRNA Control (**A**) or 25 nM of siRNA ISG15 (**B**) for 48 hrs and infected with JFH1 (m.o.i = 0.2). At the times indicated, cell extracts (4.5 mg) were incubated with anti-MAVS antibodies. In addition, cell extracts prepared at 0 hr post-infection were incubated with mouse IgG as a control of specificity (asterisk). The immunoprecipitated complexes were run on three different NuPAGE gels and blotted using Mab 71/10, anti-MAVS, anti-RIG-I or anti-TRAF3 antibodies. The expression level of each protein was controlled in the total cell extracts. (**C**)- Huh7.25.CD81 cells were incubated with JFH1 (m.o.i = 6) for 2 hrs at 37°C or at 4°C in the absence or presence of 30 µM of PRI. This drug was applied one hour before the end of the incubation time. After washing the cells twice with PBS, the cells were further incubated for 2, 4 or 6 hrs at 37°C or at 4°C in the absence or presence of PRI (added every hour). The cell extracts were processed for crosslinking of RNA to proteins before lysis, as described in Materials and Methods and different immunoprecipitations were performed with antibodies directed against PKR, RIG-I or eIF2α. After extensive washing, the presence of HCV RNA linked to the immunocomplexes was analysed by RTqPCR and the presence of the proteins was verified by Western blot. Measure of HCV RNA in the cell extracts allowed to estimate its percentage of binding to PKR as 1.09%, 0.47% and 0.25% at 2, 4 and 6 hrs post-infection respectively, and its percentage of binding to RIG-I as 0.34% at 6 hrs post-infection.

The ability of HCV to control activation of the RIG-I/MAVS pathway after induction of ISG15 through a novel PKR/MAVS pathway suggests that PKR has the possibility to bind MAVS prior to RIG-I. To determine this, we established the kinetics of these interactions, after treating the Huh7.25.CD81 cells with siRNAs targeting ISG15 prior to HCV infection. This was necessary in view of the negative control of ISG15 on RIG-I. MAVS was immunoprecipitated from the cell extracts at different times post-infection and the presence of PKR and RIG-I was examined in the immunocomplexes, as well as that of TRAF3, used as marker of activation of the MAVS signaling pathway. As expected, only PKR was able to associate with MAVS and TRAF3 in the control cells (**Figure 6A**) whereas both PKR, RIG-I and TRAF3 were found in the immunocomplexes in the absence of ISG15 (**Figure 6B**). The PKR/MAVS association took place at 4 hrs post-infection in the control cells but was observed 2 hrs earlier in the ISG15-depleted cells. Whether ISG15 plays a role in the regulation of the PKR/MAVS association remains to be determined. However, the presence of TRAF3 in association with MAVS at 2 hrs post-infection in the control cells (**Figure 6A**) correlates with its association with PKR (**Figure 5C**) which indicates that the MAVS pathway can be activated through PKR as soon as 2 hrs post infection. In ISG15 knock-down cells, the RIG-I/MAVS association occurred later at 6 hrs post-infection with an increase in TRAF3 association at 9–12 hrs post infection. Altogether, these data revealed that HCV infection triggers an earlier interaction of MAVS with PKR than with RIG-I.

Finally, we asked whether PKR was able to associate with HCV RNA and how this association can be compared to that of RIG-I. PKR and RIG-I were immunoprecipitated at 2, 4 and 6 hrs post-infection and the presence of HCV RNA was analysed in the complexes. The results showed that PKR associates with HCV RNA with best efficiency at 2 hrs post-infection. Importantly, this association was strongly inhibited in presence of PRI, thus confirming the importance of PKR DRBD in the process. In contrast, the association of HCV RNA with RIG-I was detected only at 6 hrs post-infection. Interestingly, the association between RIG-I and HCV RNA was not affected by PRI, which rules out the possibility that the initial formation of a complex between PKR and HCV RNA was a pre-requisite for the subsequent binding of RIG-I to HCV RNA. Immunoprecipitation of PKR at 1, 2, 4 and 6 hrs post-infection, in presence of an inhibitor of ribonucleases also did not lead to detection of RIG-I in the complexes (**Figure S11**). Association of HCV RNA with eIF2α, used as negative control, was not significant, thus showing the specificity of the assay (**Figure 6C**). Whether a direct interaction of PKR with HCV RNA represents the initial event leading to the MAVS-dependent induction of early ISGs remains now to be characterized. Altogether, these data reveal an earlier mobilization of PKR than RIG-I in response to HCV infection which leads to activation of a MAVS-dependent signaling pathway.

## Discussion

**Figure 7:**
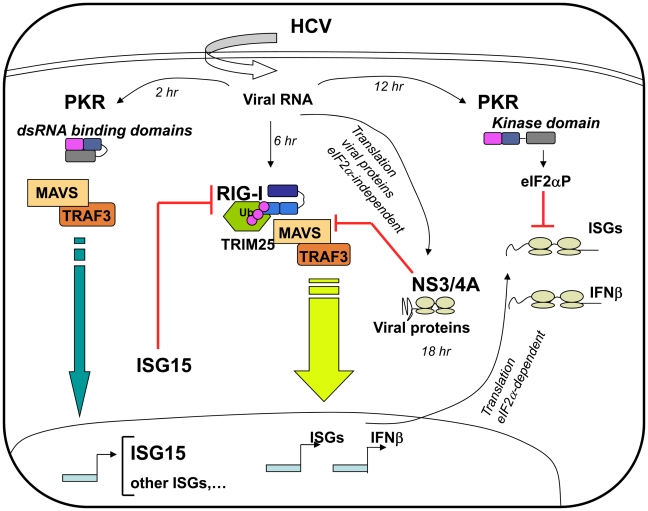
Multiple levels of control of IFN induction during HCV infection. Soon after infection, the HCV RNA is detected by the dsRNA binding domains (DRBD) of PKR ahead (2 hr) of its recognition by the RNA helicase RIG-I (6 hr). Recruitment of PKR by HCV triggers a signaling pathway that involves PKR as an adapter protein to recruit MAVS and TRAF3. This leads to a strong induction of the di-ubiquitine like protein ISG15 as well as other IRF3-dependent ISGs (Interferon-Stimulated Genes). ISG15 negatively controls the TRIM25-mediated ubiquitination (Ub) of RIG-I through an ISGylation process and thus interferes with the ability of RIG-I to recruit its downstream partners, including MAVS and TRAF3, and to induce IFNβ and ISGs. As the infection proceeds, HCV activates the eIF2α kinase function of PKR (12 hr). This leads to a transient (few hours) inhibition of general translation, including that of IFN [8] and ISGs [9] while the eIF2α-independent translation of the viral proteins proceeds unabated. At later times in the infection (18 hr), additional control of IFN induction occurs through cleavage of MAVS by the HCV NS3/4A protease, once the viral proteins have sufficiently accumulated in the cytosol [7], [27].

Hepatitis C virus can attenuate IFN induction at multiple levels in infected hepatocytes, such as through the NS3/4A-mediated MAVS cleavage [7], [27] and by using the eIF2α kinase PKR to control IFN and ISG expression at the translational level [8], [9]. Here, we have identified another process by which HCV controls IFN induction at the level of RIG-I ubiquitination through ISG15 and an ISGylation process. Importantly, we have shown that ISG15 is rapidly induced, among other ISGs, in response to HCV infection, through a novel signaling pathway that involves PKR, MAVS, TRAF3 and IRF3 but not RIG-I. In this pathway, PKR is not used for its kinase function but rather as an adapter protein with its dsRNA binding domain (DRBD) playing an essential role in this mechanism (**Figure 7**). By transcriptome analysis, we showed that HCV induces a number of ISGs in the HCV-permissive Huh7.25.CD81 cells and we confirmed the induction of two of these, ISG15 and ISG56, in other HCV-permissive cells, such as Huh7.5 and Huh7 cells. In addition, induction of ISG15 by HCV in a PKR-dependent manner was confirmed in human primary hepatocytes. The ability of HCV to trigger high expression levels of ISG15 and ISG56, as well as other ISGs, has previously been reported in models of HCV-infected chimpanzees [10], [12], [28] and in HCV-infected patients [14], [15], [16]. Induction of ISGs thus represents a general propriety of the response of the cells to HCV. In addition to this, natural variations in intra-hepatic levels of ISG15 *in vivo* may increase the susceptibility of some patients to HCV infection. The ability of HCV to control RIG-I activity through ISG15 is important to note in view of several reports which highlight the importance of a role for ISG15 in the maintenance of HCV in livers [15], [16] or in the control of HCV replication in cell cultures [17], [25]. Our data provide an explanation for the presence of ISGs at high expression levels in HCV-infected patients [14], [15], [16] and in models of HCV-infected chimpanzees [10], [12], [28] in the absence of, or with poor IFN expression.

The 15 Kda ISG15, or Interferon Stimulated Gene 15 [29], also known as ubiquitin cross reactive protein (UCRP) [30], can be conjugated (ISGylation) to more than 150 cellular protein targets [31] through the coordinated action of three E1, E2 and E3-conjugating enzymes, in a process similar but not identical to ubiquitination. While both ubiquitin and ISG15 can use the same E2 enzyme UbcH8, Ube1L functions as a specific E1 enzyme for ISG15, in spite of its 45% identity with Ube1, the E1 enzyme for ubiquitin [32]. The major E3 ligase for human ISG15 is HERC5 [33].

Interestingly, RIG-I was identified as a target for ISG15, among other IFN-induced proteins or proteins involved in IFN action [31]. However, its activity appears to be negatively controlled by ISG15 and the ISGylation process, either as shown previously after cotransfection with the ISG15 and the ISG15-conjugating enzymes [18] or as shown here, in a model of infection with HCV. Indeed, ISG15 is now emerging as playing a proviral role in case of HCV infection. Several reports now highlight the importance of a role for ISG15 in the control of HCV replication in cell cultures [17], [25] as well as in the maintenance of HCV in livers and pinpoint ISG15 as among the predictor genes of non-response to IFN therapy [14], [15], [16].

At present, we do not know at which level ISGylation regulates IFN induction in response to HCV infection. An HCV-mediated increase of ISG15 would favour preferential binding of ISG15 over that of ubiquitin to the E2 enzyme UbcH8 and hence enhance the spatio-temporal availability of UbcH8-ISG15 for HERC5 over that of UbcH8-ubiquitin for TRIM25. It may also lead to inhibition of TRIM25, through autoISGylation [21], [34], which would decrease its ability to ubiquitinate RIG-I. We showed that overexpression of HERC5 together with Ube1L, UbcH8 and ISG15 was increasing the ability of ISG15 to inhibit IFN induction by HCV (**Figure 2B**). All three enzymes Ube1L, UbcH8 and HERC5 belong to the family of genes induced by IFN and it has been reported that ISGylation is optimum in a context of IFN treatment [18], [35]. Therefore, it is tempting to speculate that elevated levels of ISG15 in some HCV-infected patients would bring the most favourable context for the virus when those patients are under IFN therapy. This would be in accord with the clinical data showing that HCV-induced high expression of ISG act as a negative predictive marker for response to IFN therapy.

It is doubtful that viruses with high IFN-inducing efficiency, such as Sendai virus may control RIG-I through ISG15 and PKR. However, viruses that avoid inducing IFN may have use of the PKR pathway. A good example might be that of Hepatitis B Virus (HBV) [36], [37], [38]. PKR expression was previously reported to be elevated in HCC liver from chronically HBV infected patients [39] and a relationship between PKR and IFN induction during HBV infection would be important to evaluate.

At present, we have established that HCV RNA interacts with PKR as soon as 2 hours post-infection. This interaction occurs prior the interaction of HCV RNA with RIG-I, which suggests that PKR may rapidly detect structures containing the incoming HCV RNA genome. Indeed, PKR has been reported to bind the dsRNA domains III and IV of HCV IRES [40] in addition to its ability to also bind 5′ triphosphorylated ss or dsRNA structures [41]. Whether PKR behaves as a pathogen recognition receptor for HCV RNA, like RIG-I, remains to be clarified. It is however clear that, in contrast to RIG-I, PKR acts here in favour of the pathogen rather than in favour of the host defense. We have established that the HCV RNA/PKR interaction depends on the first DRBD present at the N terminus of PKR and is independent on its kinase activity. The ability of PKR to serve as adapter in signaling pathways is not a total surprise since it has been previously shown to activate NF-κB through interaction of its C terminus with members of the TRAF family, such as TRAF5 and TRAF6 [42]. PKR contains also TRAF interacting motif in its N terminus [42] and an association between TRAF3 and PKR has been reported upon cotransfection in 293T cells [43]. Intriguingly, PKR was previously reported to participate in the induction of IFNβ, in association with MAVS, through activation of NF-κB or ATF-2 but not or partially IRF3; however these studies were not performed in the absence of RIG-I [44], [45], [46]. The mode of interaction between PKR, TRAF3 and MAVS, independently of RIG-I, and how it leads to a preferential induction of ISGs and not of IFNβ in response to HCV infection in contrast with the RIG-I/MAVS pathway remains to be determined. Based on our data, we propose now to divide the innate response to acute HCV infection into two phases: an early acute phase in which PKR is activated and a late acute phase that depends on RIG-I, the early phase controlling activation of the late phase. It is now essential to progress towards the generation of specific pharmaceutical inhibitors targeting PKR in order to abrogate the early acute phase to the benefit of the RIG-I-driven late phase. In a more general view, care should now be taken in the choice of compounds designed to be used as immune adjuvants, such as to be devoid of activation of the early acute PKR phase. This will ensure their efficiency as to activate properly the innate immune response through the late acute RIG-I phase.

## Methods

### Cell cultures and viruses

The culture of Huh7, Huh7.5, Huh7.25.CD81 cells, the preparation of Sendai virus stocks (≈2000 HAU/ml) and of HCV JFH1 stocks (≈6.10^4^ FFU/mL and ≈6.10^6^ FFU/mL) was as described [8], [47]. Preparation and cultures of human primary hepatocytes was as described [48]. Of note, the ability of the Huh7.25.CD81 cells to induce IFN in response to SeV without prior IFN treatment (40-fold) was not observed in our previous study [8].The ability of Sendai virus to induce IFN is related to the presence of copyback DI (Defective Interfering) genomes [49]. The higher IFN inducing ability of the novel Sendai virus stock may have come from an important accumulation of these copyback DI genomes, during its growth in chicken eggs.

### PKR inhibitors

The C16 compound [50] and the cell-permeable PRI peptide [51] were provided by Jacques Hugon. These drugs were applied (200 nM for C16 and 30 mM for PRI) one hour before the end of the 2 hr- incubation time with JFH1 and re-added to the medium after washing the cells with phosphate buffered saline (PBS). Note that PRI loses its effect very rapidly, probably through degradation in the cells, and requires to be added every hour to the cells until the end of treatment.

### Expression vectors

TRIM25 was cloned from the IFN-treated Huh7.25CD81 cells (500 U/ml IFN-α2a; Cellsciences) after RT-PCR using the forward: 5′-ATGGCAGAGCTGTGCCCCCT-3′ and reverse 5′-CTACTTGGGGGAGCAGATGG-3′ primers. The pcDNA3.1(+) vector expressing 5′HA tagged-TRIM25 (provided by D. Garcin; University of Geneva, Switzerland) was used to generate the TRIM25 P_358_L construct by site-directed mutagenesis. The IFNβ-firefly luciferase (pGL2-IFNβ) and pRL-TK Renilla-luciferase reporter plasmids were described previously [8]. The pGL3 luciferase reporter construct containing the −3 to −654 nucleotides of the ISG56 promoter was provided by N.Grandvaux [52]. The Myc-HIS-Ubiquitin construct was provided by R.Kopito (Stanford University, CA). ISG15 was cloned from IFN-treated Huh7 cells using the forward: 5′- GGATCCCATGGGCTGGGACCTGACGGTG-3′ and reverse 5′-CTCGAGCTCCGCCCGCCAGGCTCTGT-3′ primers and inserted into the pcDNA3.1(+)HA vector. The Ube1L, UbcH8 and HERC5 constructs were kindly provided by Jon M. Huibregtse [35]. The pcDNA1/AMP vector expressing PKR has been described previously [53].

### RNA-mediated interference

The siRNAs directed against PKR, MAVS, RIG-I, TRAF3 and IRF3 which were used for the experiment described in figure 5A correspond to pools of siRNA (Smartpool) obtained from Dharmacon Research, Inc. (Lafayette, CO), as well as siRNAs directed against Ube1L used in Figure 2B. Control (scrambled) siRNA and siRNA directed against PKR or ISG15, used in all other experiments, were chemically synthesized by Dharmacon (scrambled and PKR) and by EUROFINS MWG Operon (ISG15) (Text S1). The siRNAs (final concentration 25 nM or 50 nM) were transfected for 48 h using jetPRIME reagent according to the manufacturer's instructions (PolyPlus transfection TM) before transfection with other plasmids or before infection.

### Antibodies

Mab to ISG15 (clone 2.1) was a kind gift of E.Borden [54]. Mab to PKR was produced from the murine 71/10 hybridoma (Agro-bio; Fr) with kind permission of A.G.Hovanessian [55]. Other antibodies were as follows: anti-mouse IgG (Santa Cruz), anti-TRAF3 (Santa Cruz), pThr451-PKR (Alexis), MAVS (Alexis), anti-actin (Sigma), anti-pSer10-Histone H3 (Millipore), anti-HCV NS3 (Chemicon), anti-HCV core (Thermo scientific), anti-RIG-I (Alexis Biochemical Inc.), anti-TRIM25 (6105710; BD Bioscience), anti-IRF3 (Santa Cruz), anti-HA (12CA5; Roche) and anti-Myc (Santa Cruz).

### Reporter assays

Huh7.25.CD81 cells (80,000 cells/well; 24-well plates) were transfected with 40 ng of pRL-TK Renilla-luciferase reporter (Promega) and 150 ng of either pGL2-IFNβ-Firefly luciferase reporter or pISG56-luciferase reporter and processed for dual-luciferase reporter assay as reported previously [8].

### Real-time RT-PCR analysis

Total cellular RNA was extracted using the TRIZOL reagent (Invitrogen). HCV RNA was quantified by one-step RTqPCR. Reverse-transcription, amplification and real-time detection of PCR products were performed with 5 µl total RNA samples, using the SuperScript III Platinum one-step RTqPCR kit (Invitrogen) and an AbiPrism 7700 machine. For the sequence of the different primers, see Text S1. The results were normalized to the amount of cellular endogenous GAPDH RNA using the GAPDH control kit from EuroGentec. Copies number of HCV RNA may vary due to internal calibration and depending on the preparation of the viral stocks. All m.o.i were calculated using the titers expressed in FFU/ml. The IFNβ, ISG15, ISG56, Ube1L and GAPDH amplicons were quantified by a two-step RTqPCR assay as described [8].

### Transcriptome analysis

Cellular RNA was extracted and purified from the cells using RNAeasy mini kit (QIAGEN K.K., Tokyo, Japan). Comprehensive DNA microarray analysis was performed with 3D-Gene Human Oligo chip25k with 2-color fluorescence method by New Frontiers Research Laboratories, Toray Industries Inc, Kamakura, Japan as previously described [56]. In brief, each sample was hybridized with 3D-Gene chip. Hybridization signals were scanned using Scan Array Express (PerkinElmer, Waltham, MA). The scanned image was analyzed using GenePix Pro (MDS Analytical Technologies, Sunnyvale, CA). All the analyzed data were scaled by global normalization.

### Immunoprecipitation and immunoblot analysis

Cells were washed once with PBS and scraped into lysis buffer 1 (50 mM TRIS-HCl [pH 7.5], 140 mM NaCl, 5% glycerol, 1% CHAPS) that contained phosphatase and protease inhibitors (Complete, Roche Applied Science). The protein concentration was determined by the Bradford method. For immunoprecipitation, lysates were incubated at 4°C overnight with the primary antibodies as indicated and then in the presence of A/G-agarose beads (Santa Cruz Biotechnology) for 60 minutes. The beads were washed three times, and the precipitated proteins were extracted at 70°C using NuPAGE LDS sample buffer. Protein electrophoresis was performed on NuPAGE 4–12% Bis TRIS gels (Invitrogen). Proteins were transferred onto nitrocellulose membranes (Biorad), and probed with specific antibodies. Fluorescent immunoblot images were acquired and quantified by using an Odyssey scanner and the Odyssey 3.1 software (Li-Cor Biosciences) as described previously [8]. For detection of ISG15, cells were lysed in RIPA buffer (50 mM TRIS-HCl [pH 8.0]; 200 mM NaCl; 1% NP-40; 0.5% Sodium Deoxycholate; 0.05% SDS; 2 mM EDTA) and protein electrophoresis was performed on 4–20% polyacrylamide gels (PIERCE).

### Nuclear/cytoplasmic extract

Pellets from cells washed in ice-cold phosphate-buffered saline (PBS) were lysed in ice-cold cytoplasmic buffer (10 mM TRIS [pH 8.0], 5 mM EDTA, 0.5 mM EGTA, 0.25% Triton X-100) containing phosphatase and protease inhibitors. The suspension was centrifuged for 30 seconds at 14,000 g and the supernatant (cytoplasmic fraction) was transferred into microcentrifuge tubes. The nuclear pellet was resuspended in Urea buffer (8 M Urea, 10 mM TRIS [pH 7,4], 1 mM EDTA, 1 mM dithiothreitol) containing phosphatase and protease inhibitors, homogenized by vortex and boiled for 10 minutes. The protein concentration was determined by the Bradford method.

### Ubiquitination assay

Huh7.25.CD81 cells were transfected for 48 hrs with 5 µg of Myc-His-Ubiquitin expression plasmid using jetPRIME reagent. The cells were then washed in ice-cold PBS containing 20 mM N-ethylmaleimide (Sigma-Aldrich), harvested directly in Gua8 buffer (6 M guanidine-HCl, 300 mM NaCl, 50 mM Na_2_HPO_4_, 50 mM NaH_2_PO_4_ [pH 8.0]), briefly sonicated, and centrifuged at 14,000 g for 15 min at 4°C. 1/10th of the lysate was subjected to precipitation with 10% trichloroacetic acid for protein analysis in whole cell extracts. The rest of the lysate was incubated for 2 hrs with 20 µl (packed volume) of Talon resin Ni-affinity beads (Clontech) on a rotating wheel. Bound proteins were washed four times in Gua8 buffer, three times in Urea 6.3 buffer (8 M Urea, 10 mM TRIS, 0.1 M Na_2_HPO_4_, 20 mM Imidazole [pH 6.3]), and three times in cold PBS, after which they were eluted by boiling in NuPAGE LDS sample buffer. Electrophoresis was performed on 4–12% of acrylamide NuPAGE gels (Invitrogen).

### Co-precipitation protein/HCV RNA

Huh7.25.CD81 cells were incubated for 10 min in their culture medium containing 1/10 volume (Vol) of a crosslinking solution (11% Formaldehyde, 0.1 M NaCl, 1 mM Na-EDTA-[pH 8], 0.5 mM Na-EGTA-[pH 8], 50 mM HEPES [pH 8]). The reaction was stopped by addition of a solution of 0.125 M glycine in PBS [pH 8] at room temperature (RT). The cells were washed three times in ice-cold PBS containing 1000 U/ml of RNAse inhibitor (Promega), scraped in PBS and dispatched into three sets containing ½ (set 1), ¼ (set 2) and ¼ (set 3) of the cell suspension. The three sets were centrifuged for 30 seconds at 14,000 g and 4°C and the cell pellets were lysed into lysis buffer 1 containing phosphatase/protease and RNAse inhibitors (Promega) for sets 1 and 2 or into TRIZOL reagent for set 3. Cell lysates from sets 1 and 2 were then incubated at 4°C, first overnight with the appropriate primary antibodies and for 60 minutes in the presence of A/G-agarose beads (Santa Cruz Biotechnology). After the incubation period, the beads were washed four times with buffer 1. Set 1 (HCV RNA bound to immunocomplexes) and set 3 (input HCV RNA) were submitted to TRIZOL treatment and HCV RNA was quantified by one-step RTqPCR as described previously. The immunoprecipitated proteins from set 2 were extracted at 70°C using NuPAGE LDS sample buffer and analysed by immunoblot after electrophoresis on 4–12% of acrylamide NuPAGE gels (Invitrogen).

## Supporting Information

Figure S1 **Efficient induction of TRIM25 by IFN in the Huh7.25.CD81 cells.** Huh7.25.CD81 cells, seeded at 8×10^4^ cells in 24-well plates containing coverslips, were treated with 500 U/ml of IFNα for 24 hrs (IFN) or left untreated (Cont). Cells were fixed with 4% PFA and TRIM25 was detected using anti-TRIM25 antibodies (red). Nuclei are shown in blue after DAPI labelling. Microscope magnification was ×63.
(PDF)

Figure S2 **HCV controls RIG-I ubiquitination through ISG15 in the Huh7 cells.** Huh7 cells were transfected for 24 hrs with 25 nM of siRNA (Control or ISG15) and for another 24 hr with 5 µg of a His-Myc-Ubiquitin plasmid in absence or presence of 5 µg of a plasmid expressing HA-TRIM25. The cells were infected with JFH1 (m.o.i = 0.2). At the times indicated, cell extracts were processed for analysis of RIG-I ubiquitination and the expression of the different proteins in the total cell extracts. Efficiency of infection by JFH1 in the Huh7 cells was 2 log less than in the Huh7.25.CD81 cells.
(PDF)

Figure S3 **Expression of ISG15 and ISG15 conjugating enzymes inhibit IFN induction in response to SeV.** Huh7.25.CD81 cells were transfected with a plasmid expressing HA-ISG15 alone or in the presence of plasmids expressing the ISG15 conjugating enzymes Ube1L (E1), UbcH8 (E2) and HERC5 (E3). The cells were then infected with JFH1 (m.o.i = 6) for the times indicated. Stimulation of endogenous IFNβ RNA expression was determined by RTqPCR and expressed as fold induction. The degree of statistical significance is indicated by stars after calculation of the p-values (from left to right: 0.0124 and 0.0058).
(PDF)

Figure S4 **Control of efficiency of siRNA Ube1L in the Huh7.25.CD81 cells.** The Huh7.25.CD81 cells were transfected with 50 nM of siRNA directed against Ube1L for 48 hours and infected with HCV. RNA was prepared from the cells at different times post infection as indicated and expression levels of Ube1L was determined by RTqPCR.
(PDF)

Figure S5 **Modulation of PKR activation by ISG15.** Huh7.25.CD81 cells, in 100 cm^2^ plates, were transfected with siRNA Control or siRNA ISG15 or transfected with a plasmid expressing HA-ISG15 for 48 hrs and infected with JFH1 (m.o.i = 6). At the indicated times post-infection, cell extracts (2.2 mg) were processed for immunoprecipitation of PKR. The immunoprecipitated complexes were run on two different NuPAGE gels and blotted using Mab 71/10 or anti-phosphorylated PKR antibodies (PKR-P). The presence of PKR and PKR-P was revealed using the Odyssey procedure. The bands corresponding to total PKR and phosphorylated PKR were quantified using the Odyssey software and expressed as the ratio PKR-P/PKR in the absence (siISG15) and in the presence of ISG15 in the control cells (Control) or after transfection of the ISG15 expressing plasmid (HA-ISG15).
(PDF)

Figure S6 **Induction of ISG56 by Sendai virus in the Huh7.25.CD81 cells does not depend on PKR.** Huh7.25.CD81 cells were either transfected with 25 nM of siRNA Control or 25 nM siPKR for 24 hrs and infected with SeV for the times indicated. The effect of PKR silencing on the stimulation of expression of endogenous ISG56 was determined by RTqPCR and expressed as fold induction. Error bars represent the mean ±S.D for triplicates. The expression levels of ISG56 RNA at the start of infection were respectively 1.15×10^5^ copies (siControl) and 1.16×10^5^ copies (siPKR).
(PDF)

Figure S7 **Control of the efficiency of siRNA treatment in the Huh7.25.CD81 cells.** The Huh7.25.CD81 cells were transfected for 48 hrs with 50 nM Control siRNA or with the different Smartpool siRNAs as shown (50 nM siPKR; 10 nM siRIG-I; 50 nM siIRF3; 50 nM siTRAF3; 5 nM siMAVS). Total cell extracts were prepared and the expression level of each protein, as well as that of actin used as control, was revealed by immunoblot and Odyssey procedure after a run on NuPAGE gels. Under each lane, the numbers represent the quantification of the different protein bands performed using the Odyssey software.
(TIF)

Figure S8 **HCV triggers nuclear translocation of IRF3 early after infection in the Huh7.25.CD81 cells.** Huh7.25.CD81 cells, seeded at 10^5^ cells in 24-well plates containing coverslips, were infected for different times (0, 4 and 6 hours) at 37°C with JFH1 (moi = 6) or with SeV (40 HAU/ml) in the absence or in the presence of 10 ng/ml Leptomycine B (LB; Sigma), which was used here as a convenient mean to enhance the nuclear detection of IRF3 since it can interfere with nuclear export [57]. Cells were fixed with 4% PFA and IRF3 was detected using anti-IRF3 antibodies (red). Nuclei are shown in blue after DAPI labelling. The arrows show the presence of IRF3 in the nucleus. Microscope magnification was ×63.
(PDF)

Figure S9 **Induction of ISG56 by HCV in the Huh7.5 and Huh7 cells depends on PKR.** Huh7.5 or Huh7 cells were transfected with siRNA Control or siPKR (50 nM) for 48 hrs and infected with JFH1 (m.o.i = 0.2 for Huh7.5 or 10 for Huh7). At the times indicated, expression of endogenous ISG56 was determined by RTqPCR and expressed as fold induction. Error bars represent the mean ±S.D for triplicates. The expression levels of ISG56 RNA at the start of infection in the siControl cells was 1.37×10^4^ copies (Huh7.5 cells) and 1.28×10^4^ copies (Huh7 cells).
(PDF)

Figure S10 **Induction of ISG56 by HCV is specifically inhibed by the PKR inhibitor PRI.** The Huh7.25.CD81 cells were incubated with PRI or C16 and infected with JFH1 (m.o.i = 0.2) for the times indicated. RTqPCR analysis of endogenous ISG56 was determined by RTqPCR and expressed as fold induction. The expression levels of ISG56 RNA at the start of infection in the control cells was 1.97×10^4^ copies.
(TIF)

Figure S11 **The RNAse inhibitor RNAsin does not favour the formation of a RIG-I/PKR complex upon HCV infection.** Two sets of Huh7.25.CD81 cells were plated into 100 cm^2^ plates and infected with JFH1. At the times indicated, cell extracts (3.5 mg) from the two sets were processed similarly for immunoprecipitation of PKR or for incubation with mouse IgG as a control of specificity (asterisk), except that care was taken to add the RNAse inhibitor RNAsin (1000 U/ml) at all steps for the second set (+RNAsin). Detection of RIG-I, MAVS, and PKR in the complexes and in the whole cell extracts (WCE) was revealed by immunoblot using the Odyssey procedure. Detection of Actin in WCE served as loading control.
(PDF)

Table S1 **Transcriptome analysis of PKR-dependent downpregulated gene upon 12 hrs of HCV infection.** Preparation of samples was as described under Table 1. The list shows genes that were affected no more than twice by the depletion of PKR in the control cells (0.5< siPKR mock/siCt <1.6). The dependence of each of these genes in regards with PKR for their inhibition by HCV is expressed as log2 (ratio (siPKR HCV/siCt Mock)−(siCt HCV/siCt Mock) (indicated by log2*) with a cut-off of ≈2.0 fold.
(DOC)

Text S1 **Supplementary methods.**
(DOC)
