# Supplementary figures and images for: Hepatitis C Virus Reveals a Novel Early Control in Acute Immune Response
Source: PLoS Pathog. 2011 Oct 13;7(10):e1002289. doi: 10.1371/journal.ppat.1002289 (PMC3192838; doi:10.1371/journal.ppat.1002289)

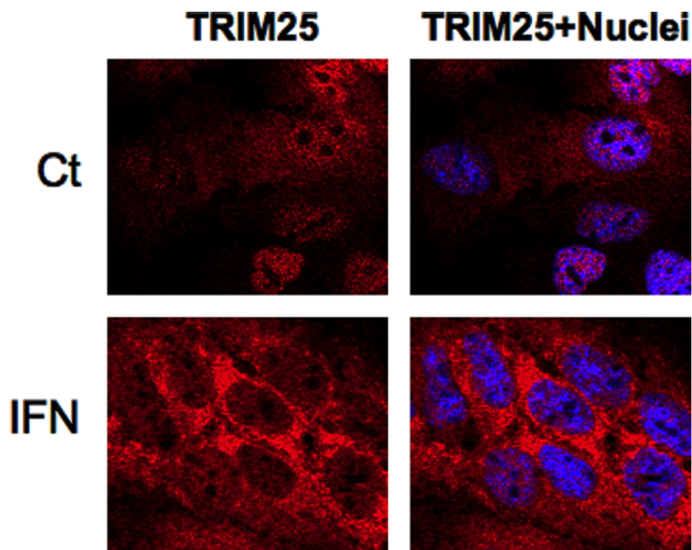

**Fig S1**

Supplement: Figure S1 — Efficient induction of TRIM25 by IFN in the Huh7.25.CD81 cells. Huh7.25.CD81 cells, seeded at 8×10^4^ cells in 24-well plates containing coverslips, were treated with 500 U/ml of IFNα for 24 hrs (IFN) or left untreated (Cont). Cells were fixed with 4% PFA and TRIM25 was detected using anti-TRIM25 antibodies (red). Nuclei are shown in blue after DAPI labelling. Microscope magnification was ×63. (PDF) [file ppat.1002289.s001.pdf]

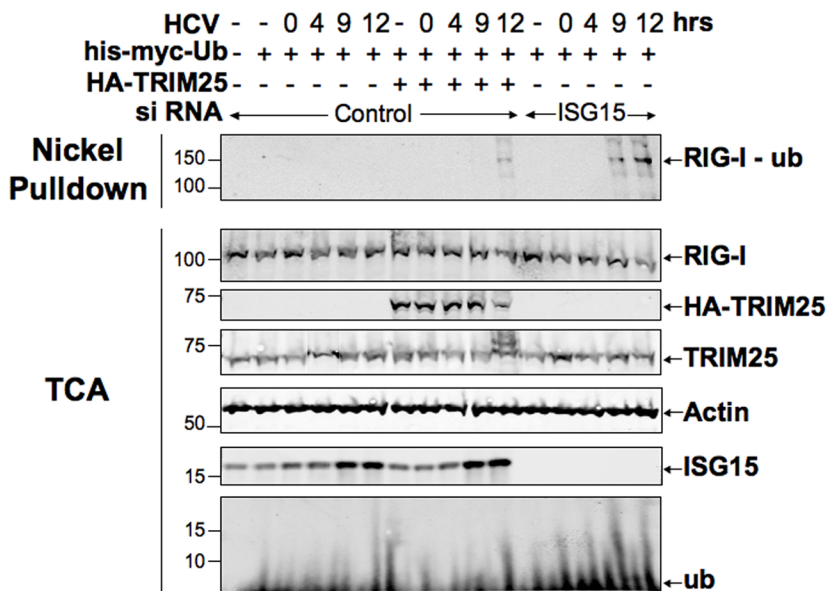

## Fig S2

Supplement: Figure S2 — HCV controls RIG-I ubiquitination through ISG15 in the Huh7 cells. Huh7 cells were transfected for 24 hrs with 25 nM of siRNA (Control or ISG15) and for another 24 hr with 5 µg of a His-Myc-Ubiquitin plasmid in absence or presence of 5 µg of a plasmid expressing HA-TRIM25. The cells were infected with JFH1 (m.o.i = 0.2). At the times indicated, cell extracts were processed for analysis of RIG-I ubiquitination and the expression of the different proteins in the total cell extracts. Efficiency of infection by JFH1 in the Huh7 cells was 2 log less than in the Huh7.25.CD81 cells. (PDF) [file ppat.1002289.s002.pdf]

IFN $\beta$  mRNA fold  
induction

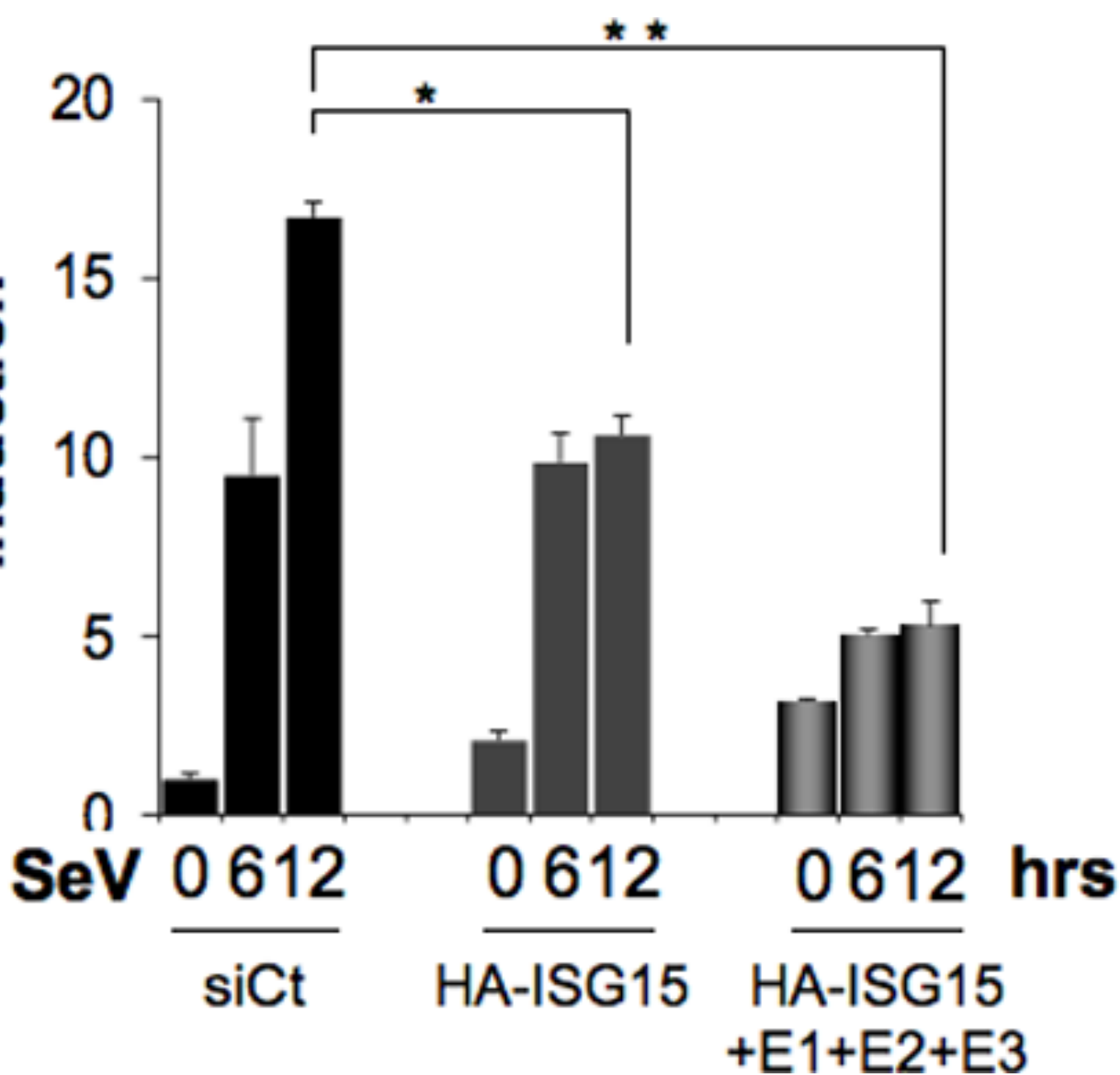

Supplement: Figure S3 — Expression of ISG15 and ISG15 conjugating enzymes inhibit IFN induction in response to SeV. Huh7.25.CD81 cells were transfected with a plasmid expressing HA-ISG15 alone or in the presence of plasmids expressing the ISG15 conjugating enzymes Ube1L (E1), UbcH8 (E2) and HERC5 (E3). The cells were then infected with JFH1 (m.o.i = 6) for the times indicated. Stimulation of endogenous IFNβ RNA expression was determined by RTqPCR and expressed as fold induction. The degree of statistical significance is indicated by stars after calculation of the p-values (from left to right: 0.0124 and 0.0058). (PDF) [file ppat.1002289.s003.pdf]

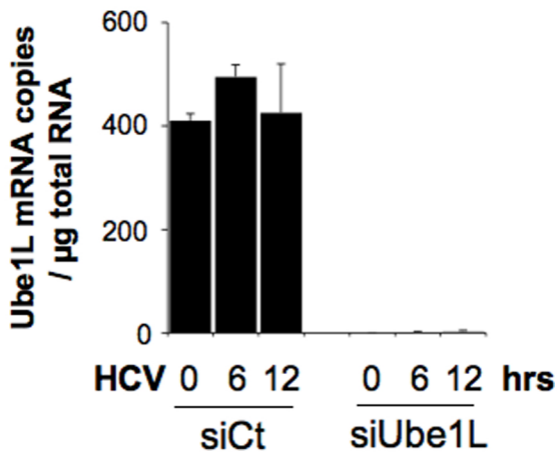

Fig S4

Supplement: Figure S4 — Control of efficiency of siRNA Ube1L in the Huh7.25.CD81 cells. The Huh7.25.CD81 cells were transfected with 50 nM of siRNA directed against Ube1L for 48 hours and infected with HCV. RNA was prepared from the cells at different times post infection as indicated and expression levels of Ube1L was determined by RTqPCR. (PDF) [file ppat.1002289.s004.pdf]

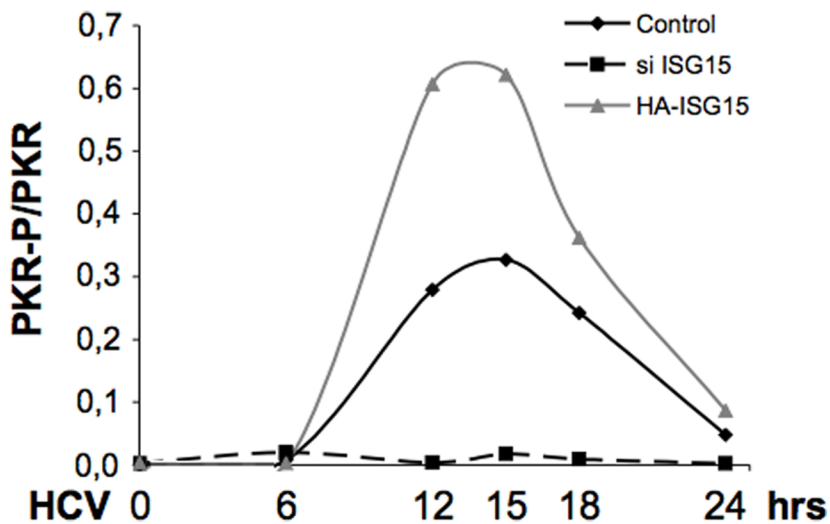

**Fig S5**

Supplement: Figure S5 — Modulation of PKR activation by ISG15. Huh7.25.CD81 cells, in 100 cm2 plates, were transfected with siRNA Control or siRNA ISG15 or transfected with a plasmid expressing HA-ISG15 for 48 hrs and infected with JFH1 (m.o.i = 6). At the indicated times post-infection, cell extracts (2.2 mg) were processed for immunoprecipitation of PKR. The immunoprecipitated complexes were run on two different NuPAGE gels and blotted using Mab 71/10 or anti-phosphorylated PKR antibodies (PKR-P). The presence of PKR and PKR-P was revealed using the Odyssey procedure. The bands corresponding to total PKR and phosphorylated PKR were quantified using the Odyssey software and expressed as the ratio PKR-P/PKR in the absence (siISG15) and in the presence of ISG15 in the control cells (Control) or after transfection of the ISG15 expressing plasmid (HA-ISG15). (PDF) [file ppat.1002289.s005.pdf]

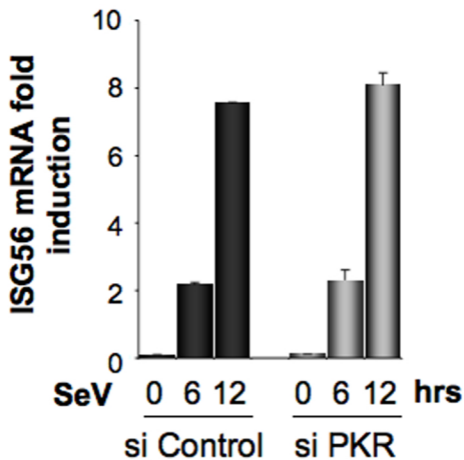

**Fig S6**

Supplement: Figure S6 — Induction of ISG56 by Sendai virus in the Huh7.25.CD81 cells does not depend on PKR. Huh7.25.CD81 cells were either transfected with 25 nM of siRNA Control or 25 nM siPKR for 24 hrs and infected with SeV for the times indicated. The effect of PKR silencing on the stimulation of expression of endogenous ISG56 was determined by RTqPCR and expressed as fold induction. Error bars represent the mean ±S.D for triplicates. The expression levels of ISG56 RNA at the start of infection were respectively 1.15×10^5^ copies (siControl) and 1.16×10^5^ copies (siPKR). (PDF) [file ppat.1002289.s006.pdf]

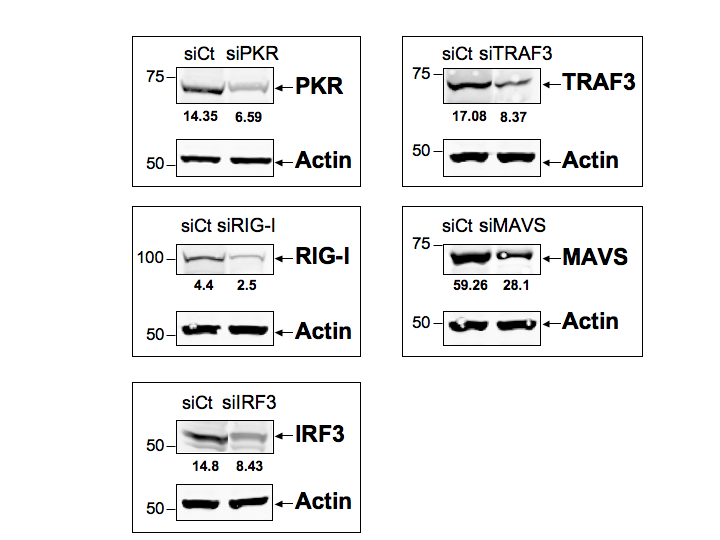

Supplement: Figure S7 — Control of the efficiency of siRNA treatment in the Huh7.25.CD81 cells. The Huh7.25.CD81 cells were transfected for 48 hrs with 50 nM Control siRNA or with the different Smartpool siRNAs as shown (50 nM siPKR; 10 nM siRIG-I; 50 nM siIRF3; 50 nM siTRAF3; 5 nM siMAVS). Total cell extracts were prepared and the expression level of each protein, as well as that of actin used as control, was revealed by immunoblot and Odyssey procedure after a run on NuPAGE gels. Under each lane, the numbers represent the quantification of the different protein bands performed using the Odyssey software. (TIF) [file ppat.1002289.s007.tif]

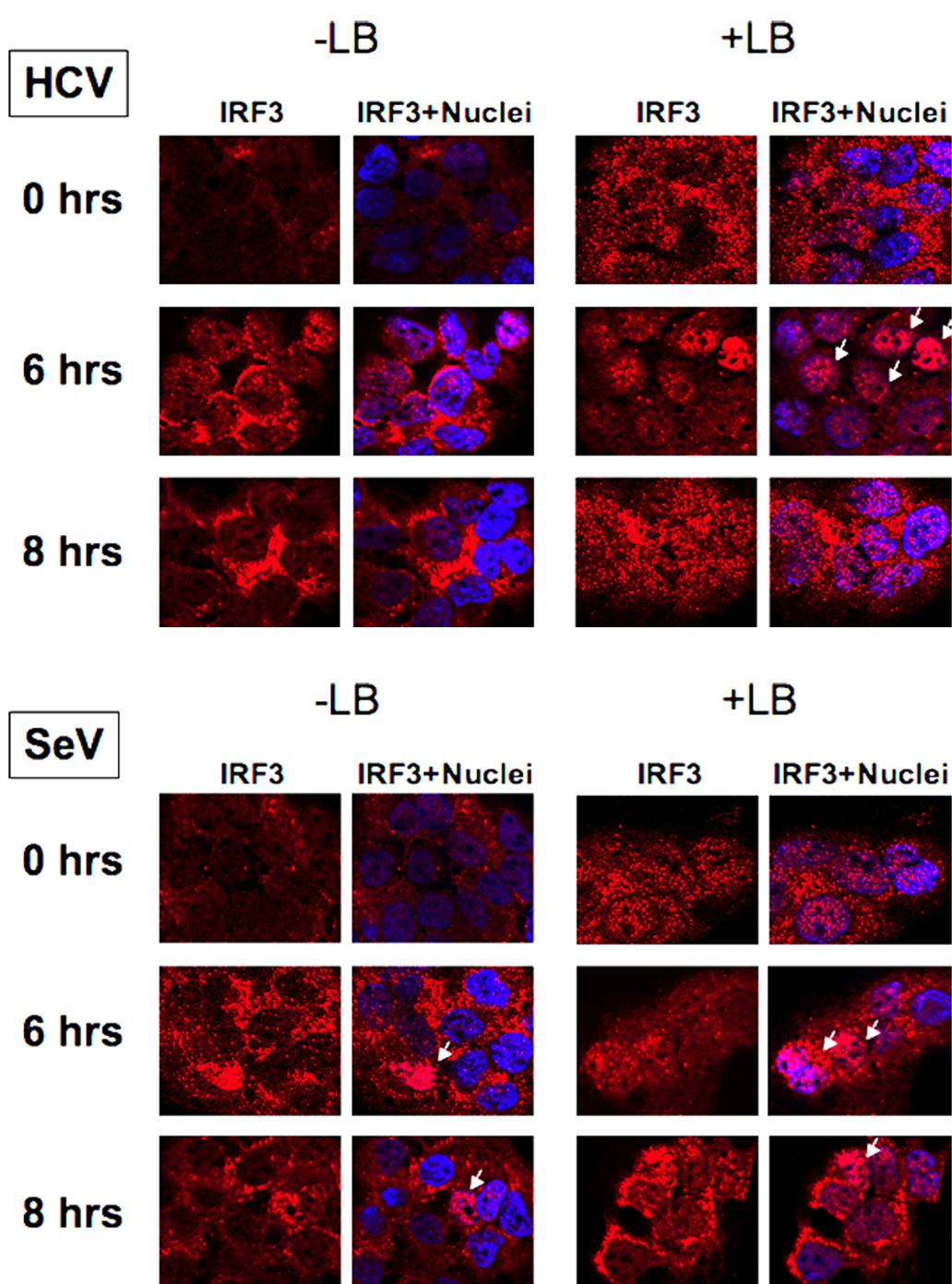

**Fig S8**

Supplement: Figure S8 — HCV triggers nuclear translocation of IRF3 early after infection in the Huh7.25.CD81 cells. Huh7.25.CD81 cells, seeded at 105 cells in 24-well plates containing coverslips, were infected for different times (0, 4 and 6 hours) at 37°C with JFH1 (moi = 6) or with SeV (40 HAU/ml) in the absence or in the presence of 10 ng/ml Leptomycine B (LB; Sigma), which was used here as a convenient mean to enhance the nuclear detection of IRF3 since it can interfere with nuclear export [57]. Cells were fixed with 4% PFA and IRF3 was detected using anti-IRF3 antibodies (red). Nuclei are shown in blue after DAPI labelling. The arrows show the presence of IRF3 in the nucleus. Microscope magnification was ×63. (PDF) [file ppat.1002289.s008.pdf]

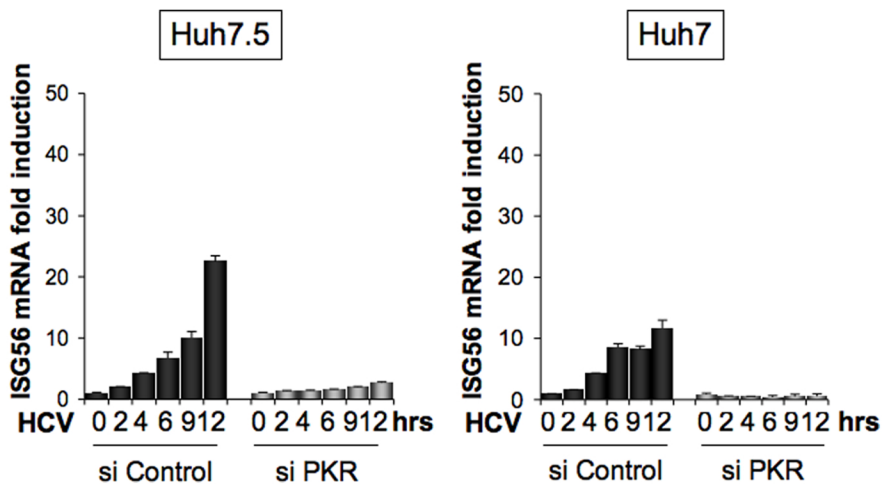

**Fig S9**

Supplement: Figure S9 — Induction of ISG56 by HCV in the Huh7.5 and Huh7 cells depends on PKR. Huh7.5 or Huh7 cells were transfected with siRNA Control or siPKR (50 nM) for 48 hrs and infected with JFH1 (m.o.i = 0.2 for Huh7.5 or 10 for Huh7). At the times indicated, expression of endogenous ISG56 was determined by RTqPCR and expressed as fold induction. Error bars represent the mean ±S.D for triplicates. The expression levels of ISG56 RNA at the start of infection in the siControl cells was 1.37×10^4^ copies (Huh7.5 cells) and 1.28×10^4^ copies (Huh7 cells). (PDF) [file ppat.1002289.s009.pdf]

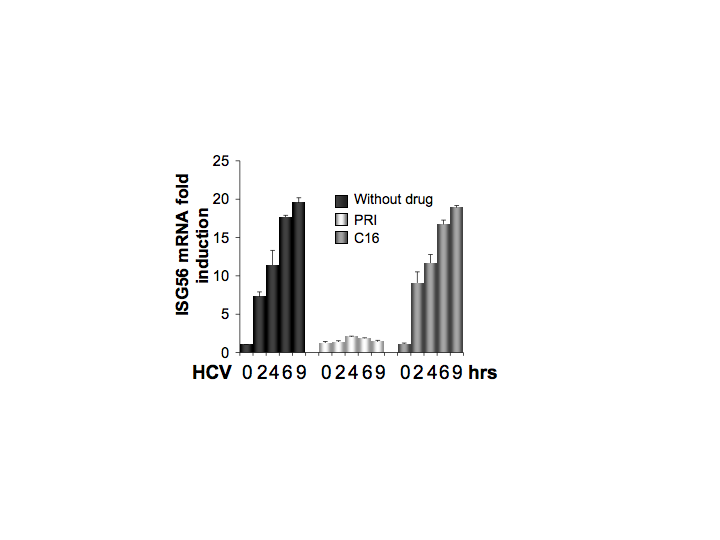

Supplement: Figure S10 — Induction of ISG56 by HCV is specifically inhibed by the PKR inhibitor PRI. The Huh7.25.CD81 cells were incubated with PRI or C16 and infected with JFH1 (m.o.i = 0.2) for the times indicated. RTqPCR analysis of endogenous ISG56 was determined by RTqPCR and expressed as fold induction. The expression levels of ISG56 RNA at the start of infection in the control cells was 1.97×10^4^ copies. (TIF) [file ppat.1002289.s010.tif]

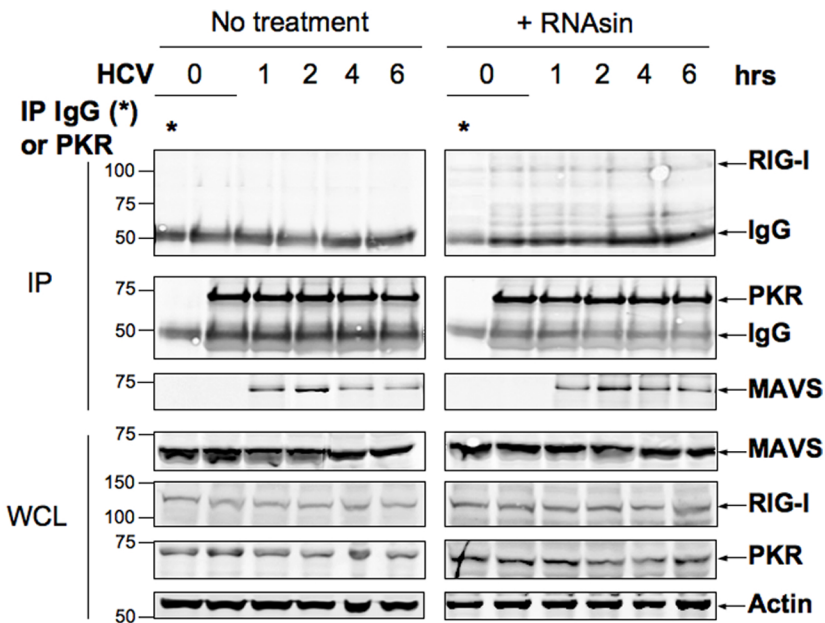

**Fig S11**

Supplement: Figure S11 — The RNAse inhibitor RNAsin does not favour the formation of a RIG-I/PKR complex upon HCV infection. Two sets of Huh7.25.CD81 cells were plated into 100 cm2 plates and infected with JFH1. At the times indicated, cell extracts (3.5 mg) from the two sets were processed similarly for immunoprecipitation of PKR or for incubation with mouse IgG as a control of specificity (asterisk), except that care was taken to add the RNAse inhibitor RNAsin (1000 U/ml) at all steps for the second set (+RNAsin). Detection of RIG-I, MAVS, and PKR in the complexes and in the whole cell extracts (WCE) was revealed by immunoblot using the Odyssey procedure. Detection of Actin in WCE served as loading control. (PDF) [file ppat.1002289.s011.pdf]
