# Supplementary material for: Hepatitis C Virus Reveals a Novel Early Control in Acute Immune Response
Source: PLoS Pathog. 2011 Oct 13;7(10):e1002289. doi: 10.1371/journal.ppat.1002289 (PMC3192838; doi:10.1371/journal.ppat.1002289)
Supplement: Table S1 — Transcriptome analysis of PKR-dependent downpregulated gene upon 12 hrs of HCV infection. Preparation of samples was as described under Table 1. The list shows genes that were affected no more than twice by the depletion of PKR in the control cells (0.5< siPKR mock/siCt <1.6). The dependence of each of these genes in regards with PKR for their inhibition by HCV is expressed as log2 (ratio (siPKR HCV/siCt Mock)−(siCt HCV/siCt Mock) (indicated by log2*) with a cut-off of ≈2.0 fold. (DOC) [file ppat.1002289.s012.doc]

**PKR-dependent down-regulated genes upon HCV infection**

| **siPRKmock/siCt** | **Name** | **Access N.** | **siCtMock** | **siCt HCV** | **siCtMock'** | **siPKRHCV** | **LOG2*** |
| --- | --- | --- | --- | --- | --- | --- | --- |
| 1,1 | Complement C7 | NM_000587 | 7,7 | 4,3 | 5,1 | 13,8 | 2,3 |
| 0,6 | RnaseA k6 | NM_005615.3 | 20,3 | 4,7 | 26,3 | 29,3 | 2,3 |
| 1 | ABCAB | NM_007168 | 9,0 | 6,6 | 4,0 | 13,0 | 2,2 |
| 1,3 | GABRA4 | NM_000809 | 13,1 | 5,4 | 13,1 | 23,6 | 2,1 |
| 0,7 | DLG2 | NM_001364 | 11,3 | 5,1 | 10,4 | 20,5 | 2,1 |
| 1,2 | none | NM_006487 | 12,8 | 6,2 | 11,4 | 23,7 | 2,1 |
| 1,1 | OPRM1 | NM_001008505 | 21,1 | 9,3 | 15,9 | 29,4 | 2,1 |
| 1,7 | DIRC2 | NM_032839 | 122,7 | 76,6 | 124,6 | 326,1 | 2,1 |
| 0,7 | NOTCH3 | NM_000435 | 17,5 | 5,5 | 14,1 | 18,3 | 2,0 |
| 1,1 | PP565 | Q8WY63 | 18,5 | 8,7 | 12,2 | 23,4 | 2,0 |
| 1,2 | MRPS6 | NM_006933 | 16,6 | 9,1 | 14,3 | 32,0 | 2,0 |
| 0,5 | GLYAT | NM_005838 | 8,5 | 4,6 | 6,9 | 15,0 | 2,0 |
| 1,6 | JAG1 | NM_000214 | 385,4 | 267,7 | 358,7 | 989 | 2,0 |
| 1,5 | B4GALNT2 | NM_153446 | 8,6 | 4,3 | 8,2 | 16,4 | 2,0 |
| 1,6 | FBN1 | NM_000138 | 37,6 | 20,7 | 36,0 | 75,5 | 1,9 |
| 1,0 | NP_775783.1 | NM_173512 | 10,8 | 4,8 | 6,6 | 11,1 | 1,9 |
| 0,9 | SPATA19 | NM_174927 | 7,9 | 4,3 | 6,0 | 11,9 | 1,9 |
