# Supplementary material for: Hepatitis C Virus Reveals a Novel Early Control in Acute Immune Response
Source: PLoS Pathog. 2011 Oct 13;7(10):e1002289. doi: 10.1371/journal.ppat.1002289 (PMC3192838; doi:10.1371/journal.ppat.1002289)
Supplement: Text S1 — Supplementary methods. (DOC) [file ppat.1002289.s013.doc]

**Hepatitis C Virus Reveals a Novel Early Control in Acute Immune Response**

Running head : HCV controls IFN induction through PKR and ISG15

Noëlla Arnaud^1^, Stéphanie Dabo^1^, Daisuke Akazawa^2^, Masayoshi Fukasawa^3^, Fumiko Shinkai-Ouchi^3^, Jacques Hugon^4^, Takaji Wakita^2^ and Eliane F.Meurs^1^*

*^1^Institut Pasteur, Hepacivirus and Innate Immunity, Paris, FR;* ^2^ *National Institute of Infectious Diseases, Department of Virology II, Tokyo, JN; ^3^ National Institute of Infectious Diseases, Department of Biochemistry and Cell Biology, Tokyo, JN; ^4^ Institut du Fer à Moulin, Inserm UMRS 839, Paris, France*

* Corresponding author

**Supplementary Methods**

**RNA-mediated interference**: Control (scrambled) siRNA, siRNA to MAVS and siRNA to PKR (GCAGGGAGUAGUACUUAAAUAUU) were synthesized by Dharmacon Research, Inc. (Lafayette, CO). siRNA to ISG15 (GCAGAUCACCCAGAAGAUU) were synthesized by Eurofins MWG Operon.

**Real-time RT-PCR Analysis:** The sequence of the different primers used for HCV RNA amplification are: 5′-TGCGGAACCGGTGAGTACA-3′ and 5′-CGGGTTGATCCAAGAAAGGA-3′, together with the internal probe 5′FAM- CGGAATTGCCAGGACGACCGG-3′TAMRA. The RNAs corresponding to IFN-β, ISG15, ISG56 and Renilla luciferase RNA were quantified by a two-step qRT-PCR assay. The reverse transcription step was performed on 1 µg of total RNA with oligodT using the AMV Reverse transcriptase (PROMEGA). Quantitative PCR was performed using an AbiPrism 7700 machine, with a SYBR GREEN PCR Master Mix (Applied BioSystemes). For the different PCRs, the following pairs of primers were used: IFN-β: 5’-TGCATTACCTGAAGGCCAAG-3’ and 5’-AAGCAATTGTCCAGTCCCA-3’; ISG15: 5’-ccacctgaagcagcaagtga-3’ and 5’-cgcaggcgcagattcatgaa-3’; ISG56: 5’-ggacaggaagctgaaggag-3’ and 5’-agtgggtgtttcctgcaa-3’; Renilla luciferase: 5’-atcatccctgatctgatcgg-3’ and 5’-ggagtagtgaaaggccagac-3’. Ube1L: 5’-ACCCAGACTTACAGGTGAT-3’ and 5’-TTCAGATAGTGGGTGCAACG-3’; GAPDH 5’-GGTCGGAGTCAACGGATTTG-3’ and 5’-ACTCCACGACGTACTCAGCG-3’.
